# Enabling Aqueous Phase Long‐lived Deep‐blue Phosphorescence With Layered Double Hydroxide

**DOI:** 10.1002/advs.202413896

**Published:** 2024-12-31

**Authors:** Qian Chen, Peisheng Cao, Peng Wu

**Affiliations:** ^1^ Analytical & Testing Center Chengdu 610064 China; ^2^ College of Chemistry Sichuan University Chengdu 610064 China

**Keywords:** Aqueous phase RTP, layer double hydroxide, long‐lived blue phosphorescence

## Abstract

Aqueous‐phase phosphors are of utmost importance for a myriad of applications. However, the emission wavelengths of the current aqueous organic room‐temperature phosphorescent (RTP) materials are limited to green and red bands, while the blue part is rarely reported, thus limiting the development of a full‐color RTP system. Theoretically, carboxylated benzene is expected to be blue phosphorescence‐emissive, but only green phosphorescence is observed in solid, due to the strong intermolecular π–π stacking that decreases the energy gap. Herein, the use of water‐dispersible layered double hydroxide (ZnAl‐LDH) is proposed for isolating Ph‐(COOH)_n_ and the distance between the adjacent chromophores is confirmed to be larger (≈7.0 Å) than the threshold of π–π interaction. Deep‐blue phosphorescence with a lifetime over 0.1 s, and a maximum luminescence quantum yield of 42%, is harvested in the aqueous phase. The long‐lived deep‐blue phosphorescence is successfully explored for high‐temperature display and luminescent dyes.

## Graphical abstract

Aqueous phase deep‐blue RTP is first achieved through isolating Ph‐(COOH)_n_ (*n* = 1–6) with layered double hydroxides (LDH), with the shortest emission wavelength at 404 nm, lifetime over 0.1 s, and maximum luminescence quantum yield of 42%.

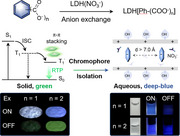

## 1 Introduction

**Scheme 1:**
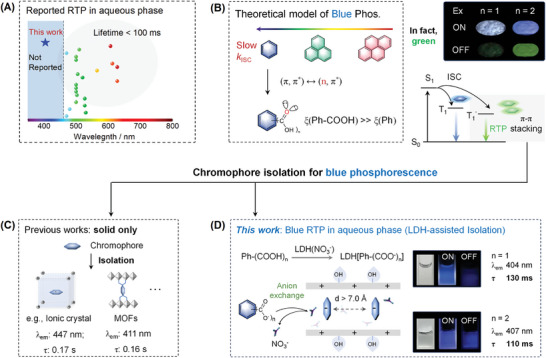
Harvesting blue phosphorescence from Ph‐(COOH)_n_: A) reported RTP in aqueous phase; B) theoretical model of blue phosphorescence; C) previous works using ionic crystal or MOFs for isolating of Ph‐(COOH)_n_ chromophores, but mostly in solid phase; and D) the proposed LDH‐assisted isolation of Ph‐(COOH)_n_ chromophores for blue RTP in aqueous phase, with photographs showing the deep blue RTP from benzoic acid (*n* = 1) and iso‐phthalic acid (*n* = 2).

Aqueous phase room temperature phosphorescence (RTP) materials are appealing for applications in lighting, sensing, and imaging.^[^ ^1^ ^]^ However, the luminescence efficiency of solution phosphors is generally largely lower than their solid counterparts, due to severe phosphorescence quenching from molecular motions and dissolved oxygen in solution.^[^ ^2^ ^]^ To suppress these nonradactive relaxations, some rigid organic nanoparticles and supermolecular hosts (e.g., cyclodextrin and cucurbituril) have been constructed to induce aqueous RTP.^[^ ^3^ ^]^ Generally, the phosphorescence emission of the currently reported aqueous‐phase RTP materials falls into the range of 450–650 nm (**Scheme** **1**A and Table S1, and Figure S1, Supporting Information), thus limiting the development of full‐color RTP‐based displaying.^[^ ^4^ ^]^ Besides, their lifetimes were generally lower than 100 ms and thus were difficult to be compatible with eye‐catchable sensing. Therefore, simple, highly efficient, and long‐lived blue phosphorescence in the aqueous phase still remains a great challenge to date.

In principle, the luminescence wavelength of organic luminophores is generally directly proportional to the size of the conjugation systems,^[^ ^5^ ^]^ and benzene is thus theoretically applicable to achieve blue RTP (Scheme 1B). However, the spin‐orbit coupling (SOC) constant of benzene is intrinsically low and difficult to facilitate efficient intersystem crossing (ISC), thus leading to extremely low phosphorescence of benzene (only cryogenically observable). According to the El‐Sayed rule, introducing the n orbital to hybridize with the ππ* transition can significantly increase the SOC constant (ξ).^[^ ^6^ ^]^ In this manner, grafting heteroatoms with lone electron pairs (e.g., O and N) onto the benzene ring (e.g., carboxyl groups) is expected to achieve blue phosphorescence. However, the phosphorescence of Ph‐(COOH)_n_ (*n* = 1–6), although more efficient than benzene, generally falls into the range of 500–600 nm (only in solid‐state), due to strong intermolecular π–π stacking that further decreases the energy gap (Scheme 1B). Therefore, separating a single benzene‐based chromophore from the stacked form is an effective way for blue RTP. For example, blue RTP was successfully harvested from isolating Ph‐(COOH)_n_ with ionic crystal (*n* = 4, 6)^[^ ^7^ ^]^ and metal‐organic frameworks (MOFs, *n* = 2),^[^ ^8^ ^]^ but still in the solid state (Scheme 1C).^[^ ^9^ ^]^

Herein, through isolating Ph‐(COOH)_n_ (*n* = 1–6) with layered double hydroxides (LDH), aqueous phase long‐lived deep‐blue RTP was achieved (Scheme 1D). LDH is a class of synthetic clays with brucite‐like cationic layer and hydrated hydrophilic interlayer, thus highly water dispersible.^[^ ^10^ ^]^ Upon anion exchange with LDH in the aqueous phase, Ph‐(COOH)_n_ could be intercalated into the layer of LDH, in which the chromophore isolation was facilitated through surrounding with multiple NO_3_^−^. Besides, the LDH matrix could provide a strict and rigid barrier to inhibit molecular motions and water‐/oxygen‐induced phosphorescence quenching. It should be noted that using LDH as a matrix to facilitate RTP was already reported, but still green RTP was harvested.^[^ ^11^ ^]^ Here, for the first time, deep‐blue RTP with a lifetime larger than 0.1 s was efficiently (maximum QY: ≈42%) harvested in the aqueous phase (Scheme 1A).

## 2 Results and Discussion

### 2.1 Blue Phosphorescence from LDH(Ph‐COO^−^) and LDH[Ph‐m(COO^−^)_2_] in Aqueous Phase

**Figure 1:**
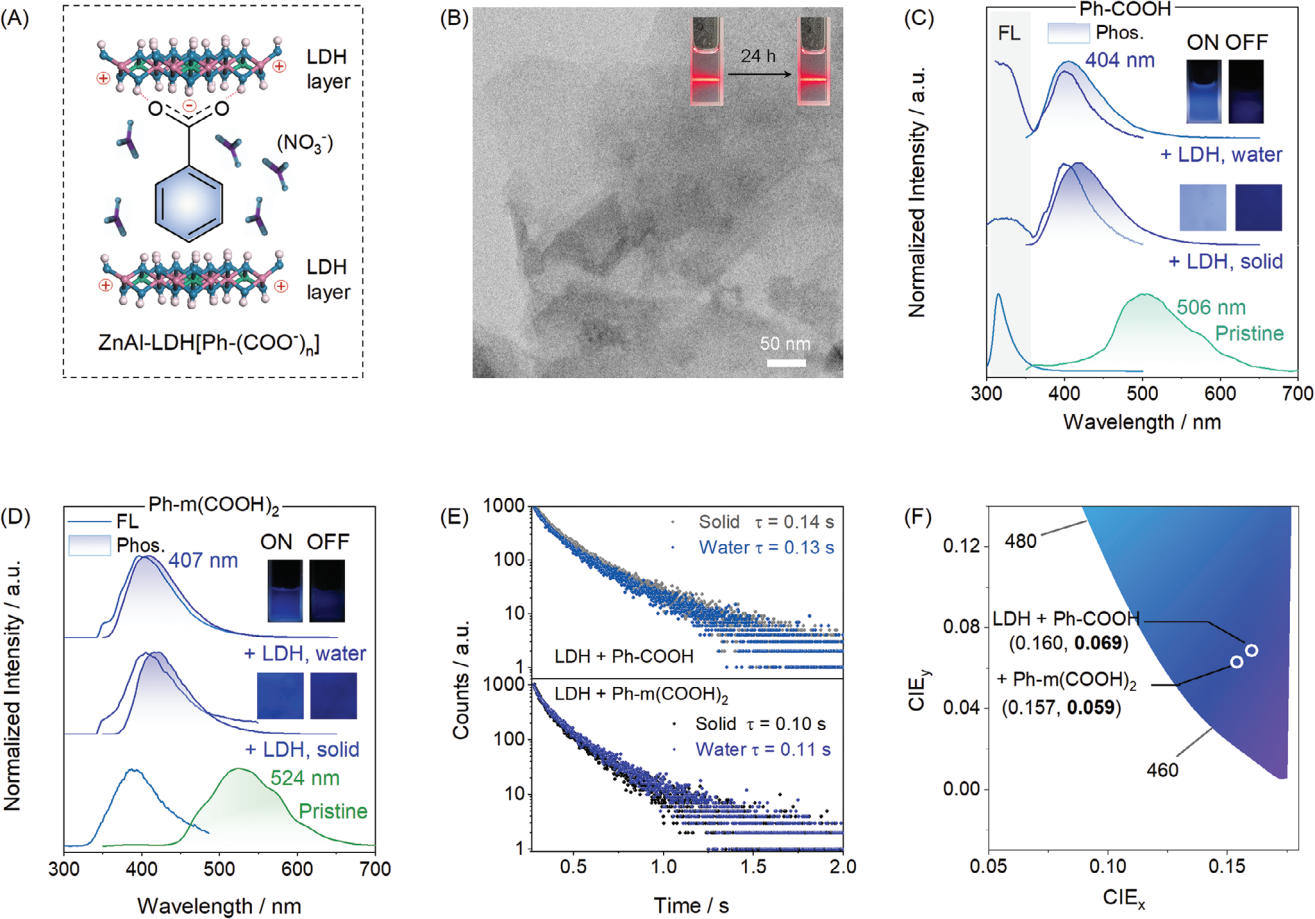
Deep‐blue phosphorescence emission from ZnAl‐LDH[Ph‐(COO^−^)_n_] (*n* = 1 and 2) in aqueous phase: A) schematic illustration of ZnAl‐LDH‐encapsulated ZnAl‐LDH[Ph‐(COO^−^)_n_] (*n* = 1); B) transmission electron microscopic and photographic images of ZnAl‐LDH[Ph‐m(COO^−^)_2_]; C) steady‐state and delayed (Δt = 10 ms) emission spectra of pristine Ph‐COOH (solid) and ZnAl‐LDH(Ph‐COO^−^) (solid and aqueous phase, λ_ex_ = 280 nm, with afterglow images shown inset); D) steady‐state and delayed (Δt = 10 ms) emission spectra of pristine Ph‐m(COOH)_2_ (solid) and ZnAl‐LDH[Ph‐m(COO^−^)_2_] (solid and aqueous phase, λ_ex_ = 280 nm, with afterglow images shown inset); E) lifetime of ZnAl‐LDH(Ph‐COO^−^) and ZnAl‐LDH[Ph‐m(COO^−^)_2_] (λ_ex_ = 280 nm) in solid and aqueous phase; F) CIE 1931 coordinates of phosphorescence emission for ZnAl‐LDH(Ph‐COO^−^) and ZnAl‐LDH[Ph‐m(COO^−^)_2_] in aqueous phase.

To show the LDH‐assisted isolation of chromophores, here ZnAl‐LDH(NO_3_^−^) and Ph‐(COOH)_n_ (*n* = 1 and 2) were chosen for study. Ph‐COOH and Ph‐m(COOH)_2_ were well intercalated into the interlayer of ZnAl‐LDH to form ZnAl‐LDH[Ph‐(COO^−^)_n_] (**Figure** **1**A), as evidenced by the X‐ray diffraction (XRD) (Figure S4, Supporting Information) and Fourier transform infrared spectroscopy (FT‐IR, Figure S5, Supporting Information) characterizations. The as‐synthesized ZnAl‐LDH[Ph‐m(COO^−^)_2_] showed thin‐flake morphology and could be well‐dispersed in aqueous solution (Figure 1B). For the obtained ZnAl‐LDH(Ph‐COO^−^), blue phosphorescence peaked at ≈404 nm in both solid and aqueous phases was observed (Figure 1C), which blue shifts ≈100 nm as compared with pristine Ph‐COOH solid. The phosphorescence lifetime of the solution RTP was determined to be ≈0.13 s (Figure 1E), thus facilitating eye‐catchable blue afterglow emission (after ceasing the excitation) in the aqueous phase (Figure 1C). For ZnAl‐LDH[Ph‐m(COO^−^)_2_], similar long‐lived blue phosphorescence at 407 nm in the aqueous phase was observed (Figure 1D,E and Figure S6, Supporting Information). Substitution of Ph‐m(COOH)_2_ with o‐phthalic acid (o‐PA) and p‐phthalic acid (p‐PA) also yielded similar long‐lived blue RTP (Figure S7, Supporting Information). Most importantly, an RTP lifetime higher than 0.1 s was harvested from LDH[Ph‐(COO^−^)_n_] (*n* = 1, 2) in the aqueous phase, and was almost identical to those in the solid state (Figure 1E). Moreover, the RTP profile of LDH/Ph‐m(COOH)_2_ almost resembled their cryogenic phosphorescence in diluted ethanol solution (77 K, Figure S8, Supporting Information), indicating the luminophore of blue RTP in LDH/Ph‐(COOH)_n_ was from the isolated Ph‐(COOH)_n_.

According to the CIE coordinate diagram (1931), the RTP color of the LDH[Ph‐(COO^−^)_n_] (*n* = 1, 2) solution could be assigned to deep‐blue emission (CIE_y_ <0.08) (Figure 1F). Although blue RTP was successfully achieved with previous isolation‐based strategies,^[^ ^7^, ^8^, ^9^ ^]^ achieving CIE y lower than 0.08 was seldom reported. Besides, the isolation of Ph‐COOH was not reported. These comparisons clearly highlighted the high performance of LDH in isolating all Ph‐(COOH)_n_ (*n* = 1–6). Probably, the dense metal hydroxides [Zn(OH)_2_ and Al(OH)_3_] as well as the residue NO_3_^−^ could efficiently prevent the typical phosphorescence quenching from H_2_O and O_2_ (Figure S9, Supporting Information). Notably, LDH(Ph‐COO^−^) and LDH[Ph‐m(COO^−^)_2_] exhibited photoluminescence quantum yield of 2.50% and 5.88% in the aqueous phase (Figure S10, Supporting Information), respectively, indicating the LDH‐assisted isolation strategy was not only effective but also efficient in deep‐blue RTP inducing.

### 2.2 Anion Exchange for Chromophore Inclusion

**Figure 2:**
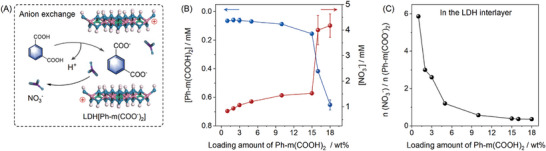
The anion exchange process: A) schematic illustration of the anion exchange between ZnAl‐LDH(NO_3_^−^) and Ph‐m(COOH)_2_; B) Ph‐m(COOH)_2_ loading amount‐dependent concentration change of NO_3_^−^ and Ph‐m(COOH)_2_ in supernatant; and C) Ph‐m(COOH)_2_ loading amount‐dependent molar ratio change of NO_3_^−^ and Ph‐m(COO^−^)_2_ in the interlayer of LDH.

According to the previous knowledge of LDH,^[^ ^12^ ^]^ the intercalation of Ph‐(COOH)_n_ into ZnAl‐LDH was probably via an anion exchange process (**Figure** **2**A). For confirmation, Ph‐m(COOH)_2_ was chosen as the model here. Theoretically, for the anion exchange between ZnAl‐LDH(NO_3_^−^) and Ph‐m(COOH)_2_, NO_3_^−^ in the interlayer of LDH will be released to the solution. Meanwhile, Ph‐m(COOH)_2_ needs to be ionized (release H^+^) to form the anionic form and balance the positive charge of the metallic hydroxide layer of ZnAl‐LDH (Figure 2A). As shown in Figure 2B and Figure S11 (Supporting Information), upon increasing the loading concentrations of Ph‐m(COOH)_2_ from 1 to 15wt.%, the amounts of the supernatant Ph‐m(COOH)_2_ were very low, but those of NO_3_^−^ increased gradually, clearly indicating the anion exchange process. The maximum loading was determined to be 15wt.%, as boosted increases of both Ph‐m(COOH)_2_ and NO_3_^−^ emerged in the supernatant when the loading amount further increased, probably due to the collapse of LDH in a concentrated solution of Ph‐ m(COOH)_2_. Simultaneous monitoring of the pH change of the solution also confirmed the anion exchange process and the collapse of LDH (Figure S12, Supporting Information).

The amounts of NO_3_^−^ and Ph‐m(COO^−^)_2_ in the resultant ZnAl‐LDH[Ph‐m(COO^−^)_2_] were measured after acid dissolution of the products (after extensive washing and vacuum‐drying). It turned out that the mole ratios of NO_3_^−^ and Ph‐m(COO^−^)_2_ in LDH interlayer decreased along with the increase of Ph‐m(COOH)_2_ loading (Figure 2C and Figure S13, Supporting Information), which further confirmed the anion exchange process.

### 2.3 Mechanism of the Deep‐Blue Phosphorescence

**Figure 3:**
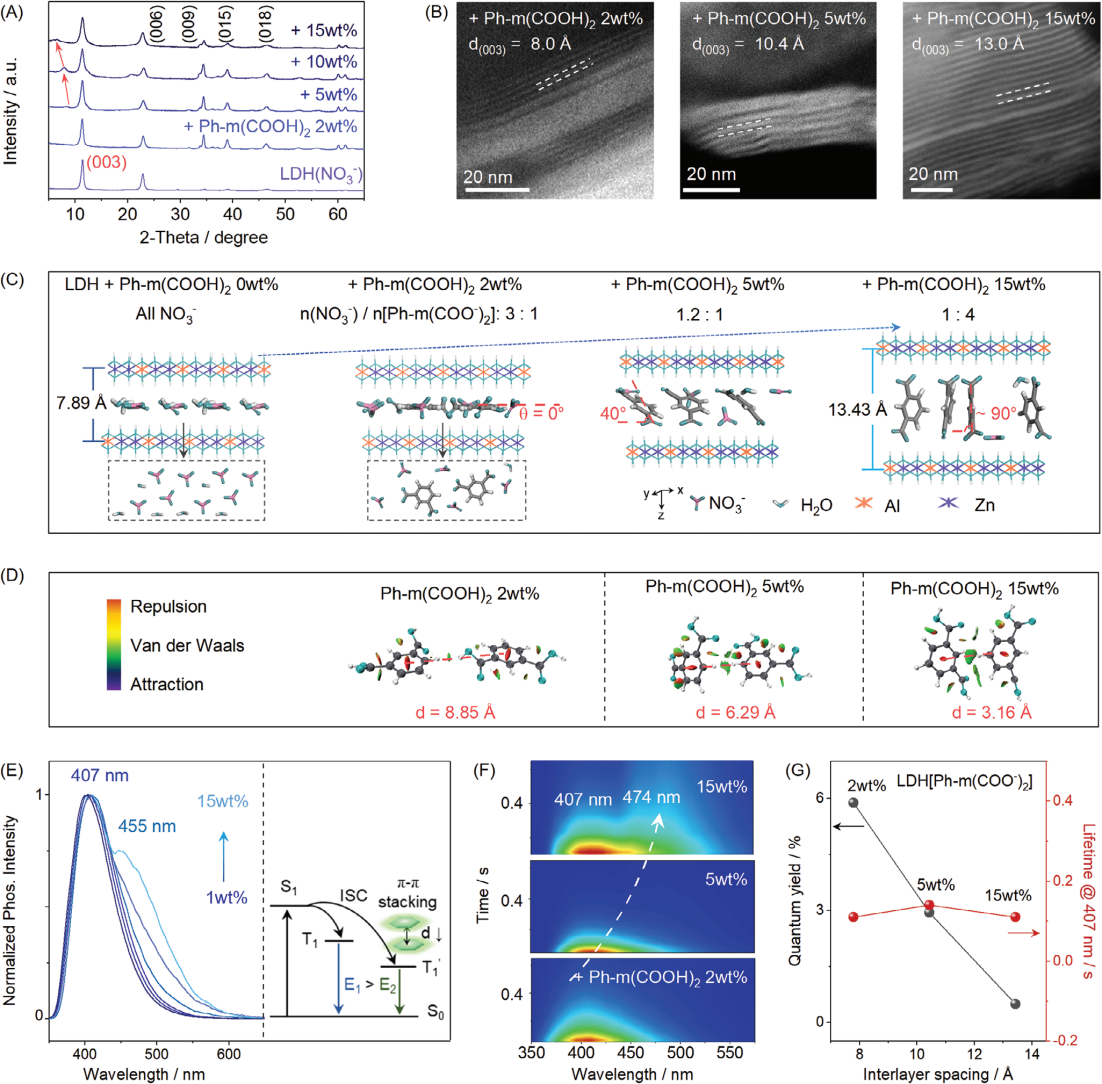
Mechanism of deep‐blue RTP from LDH[Ph‐m(COO^−^)_2_]: A) XRD patterns of ZnAl‐LDH(NO_3_^−^) and LDH[Ph‐m(COO^−^)_2_] with increasing loading amounts of Ph‐m(COOH)_2_; B) HR‐TEM images and lattice fringe spacing for LDH[Ph‐m(COO^−^)_2_] (2, 5 and 15wt.%); C) structure evolution from pristine ZnAl‐LDH(NO_3_^−^) to LDH[Ph‐m(COO^−^)_2_] (2, 5 and 15wt.%) as modeled with Vienna ab‐initio; D) the distribution of RDG iso‐surface maps for LDH[Ph‐m(COO^−^)_2_] from 2 to 15wt.%; E) the delayed (Δt = 10 ms) emission spectra of LDH[Ph‐m(COO^−^)_2_] in aqueous phase (1 mg mL^−1^, λ_ex_ = 280 nm) and the luminescence process of phosphorescence emission for isolated and aggregated phosphors; and F) TRES images of LDH[Ph‐m(COO^−^)_2_] (loading amount: 2, 5 and 15wt.%) in aqueous phase (λ_ex_ = 280 nm); and G) the relationship between the layer spacing and the lifetime and the quantum yields of LDH[Ph‐m(COO^−^)_2_].

It is evident that isolation of the chromophores is critical for the deep‐blue phosphorescence. Therefore, the distribution of Ph‐m(COO^−^)_2_ in ZnAl‐LDH was analyzed. According to the XRD patterns, for low loading of Ph‐m(COOH)_2_ (1–3wt.%), similar characteristic peaks as those of ZnAl‐LDH were obtained (Figure S14, Supporting Information). However, up to 5wt.% of Ph‐m(COOH)_2_, a new peak in the low angle region (<10°) appeared, and shifted to an even lower region upon further increasing the loading amounts (**Figure** **3**A). According to the knowledge from previous works,^[^ ^13^ ^]^ such a peak could be ascribed to the (003) crystal plane that reflects of the spacing between the interlayer of LDH. On the basis of the Bragg's law, the interlayer spacing distances of ZnAl‐LDH changed from 7.89 to 13.43 Å in the (003) direction with increasing the loading of Ph‐m(COOH)_2_ from 1 to 15wt.% (Figure S15, Supporting Information). Such results confirmed well with those observed by high‐resolution transmission electron microscopy (HRTEM, Figure 3B).

On the basis of the molar ratios of NO_3_^−^ and Ph‐m(COO^−^)_2_ in the interlayer of ZnAl‐LDH (Figure 2C) and the interlayer spacing distances (Figure 3A), the structure of LDH[Ph‐m(COO^−^)_2_] were simulated with Vienna ab‐initio. As shown in Figure 3C, for low loading of Ph‐m(COOH)_2_, the chromophores were horizontally distributed in the interlayer along the xy plane, which was isolated by the surrounding NO_3_^−^ (intermolecular distance: 8.85 Å, Figure 3C and Figure S16, Supporting Information) with weak interaction between chromophores (Figure 3D). When the loading of Ph‐m(COOH)_2_ reached 5wt.%, the interlayer spacing of LDH increased to 10.76 Å and partial of the chromophore molecules overturned in a vertical direction ≈40° (Figure 3C and Figure S17, Supporting Information). Meanwhile, the intermolecular distance was reduced to ≈6.4 Å. Up to 15wt.% loading, Ph‐m(COO^−^)_2_ molecules were almost vertically distributed in the ZnAl‐LDH interlayer (Figure 3C and Figure S18, Supporting Information), with the shortest intermolecular distance of ≈3.16 Å. Considering the threshold for identification of π–π interaction (3.8 Å),^[^ ^14^ ^]^ 5wt.% loading should be the critical point for the complete isolation of the chromophore molecules.

The above theoretical results were further confirmed spectroscopically. As shown in Figure 3E, upon increasing the loading of Ph‐m(COOH)_2_, the resultant RTP spectra slightly but gradually broadened, and eventually, a new peak (≈455 nm) besides the initial one (407 nm) emerged. Time‐resolved emission spectra (TRES) confirmed different lifetimes of these two peaks (Figure 3F), the longer wavelength of which may be ascribed to the π–π stacking‐induced new emission center (Figure 3E). As the interlayer spacing of LDH increased from 7.79 to 13.43 Å, the RTP lifetime of the isolated Ph‐m(COO^−^)_2_ kept the same, but the quantum efficiency decreased considerably, due to the decreased number of luminescent single chromophore (isolated → aggregated, Figure 3G). Therefore, the 407 nm peak could thus be attributed to the emission from isolated Ph‐m(COO^−^)_2_, which was assisted by ZnAl‐LDH.

### 2.4 Universality of Blue RTP Emission from LDH[Ph‐(COOH)_n_] in Aqueous Phase

**Figure 4:**
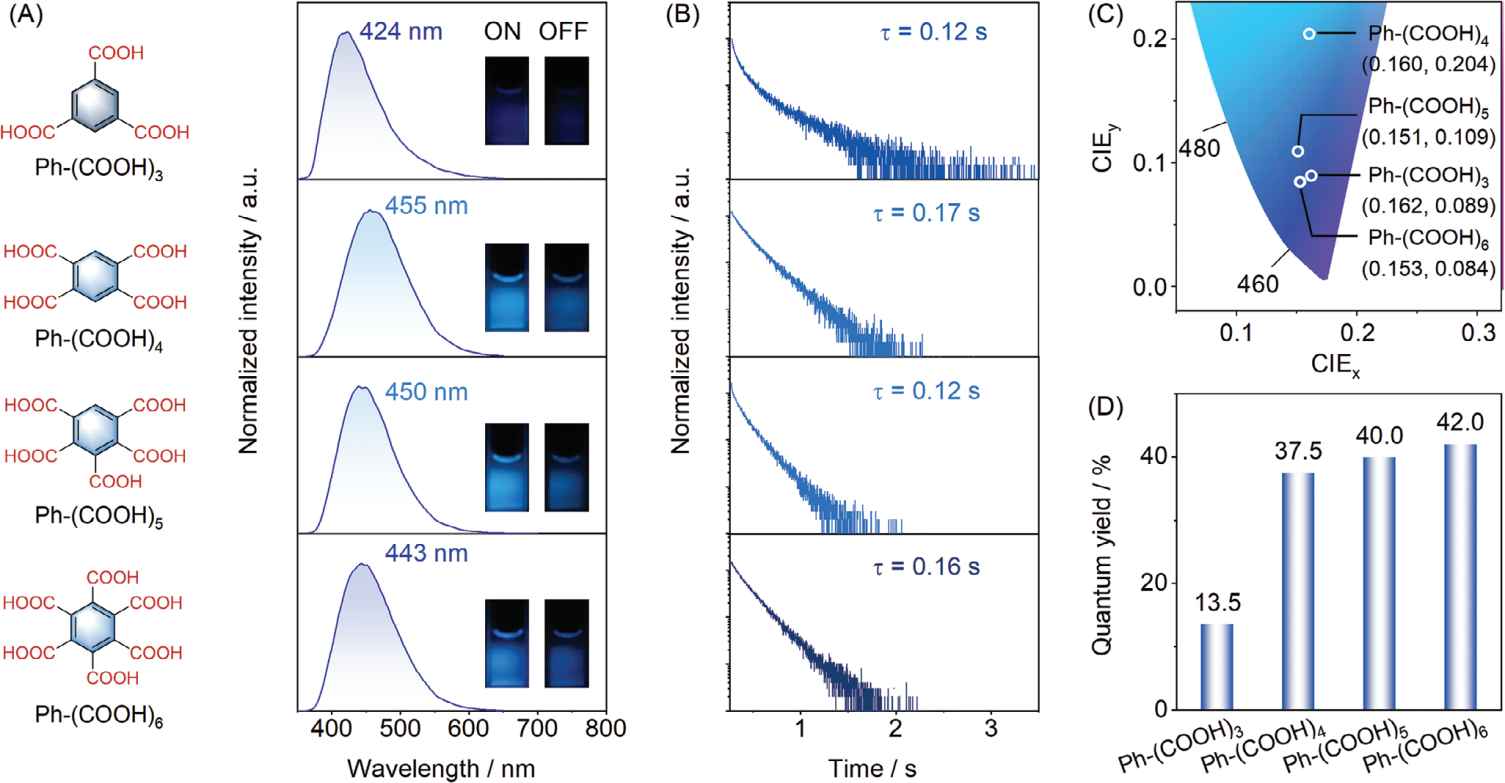
Universality exploration of deep‐blue phosphorescence from ZnAl‐LDH[Ph‐(COO^−^)_n_] (*n* = 3–6): A) the chemical structures of Ph‐(COOH)_n_ and delayed (Δt = 10 ms) emission spectra of ZnAl‐LDH[Ph‐(COO^−^)_n_] in the aqueous phase (1 mg mL^−1^, λ_ex_ = 280 nm); B) lifetime of ZnAl‐LDH[Ph‐(COO^−^)_n_] in the aqueous phase (λ_ex_ = 280 nm); C) CIE 1931 coordinates of ZnAl‐LDH[Ph‐(COO^−^)_n_] in the aqueous phase (λ_ex_ = 280 nm); D) quantum yield of ZnAl‐LDH[Ph‐(COO^−^)_n_] in the aqueous phase.

The LDH‐assisted isolation was proved to be universal for all carboxylated benzenes to yield highly efficient blue RTP in the aqueous phase. Besides the above Ph‐COOH and Ph‐(COOH)_2_, another four analogs with varied numbers of carboxyl groups, namely tribenzonic acid [Ph‐(COOH)_3_], pyromellitic acid [Ph‐(COOH)_4_], benzenepentacarboxylic acid [Ph‐(COOH)_5_], and mellitic acid [Ph‐(COOH)_6_], were also intercalated into the interlayer of ZnAl‐LDH (**Figure** **4**A and Figures S19–S21, Supporting Information). As can be seen from Figure 4A, all the as‐synthesized phosphors exhibited blue‐shifted emission when compared with the pristine Ph‐(COOH)_n_ (Figure S22 and Table S4, Supporting Information), and were confirmed with CIE coordinates (Figure 4C). Blue afterglow could be eye‐catchable (Figure S23, Supporting Information) and all the lifetimes of ZnAl‐LDH[Ph‐(COO^−^)_n_] were also second‐scale (Figure 4B). Moreover, the delayed spectra and afterglow of ZnAl‐LDH[Ph‐(COO^−^)_n_] in aqueous media almost resembled those of Ph‐(COOH)_n_ in dilute ethanol solution at 77 K (Figure S24, Supporting Information), thus confirming the successful isolation assisted by ZnAl‐LDH. Notably, a maximum quantum efficiency of 42% was observed for ZnAl‐LDH[Ph‐(COO^−^)_6_] (Figure 4D), indicating the high performance of the LDH‐assisted isolation approach. It should be noted that for blue RTP, the highest quantum efficiency of 98.6% was reported in the solid state. The detrimental effect of H_2_O on RTP thus seems unavoidable.

### 2.5 Applications of the Resultant Blue Afterglow

**Figure 5:**
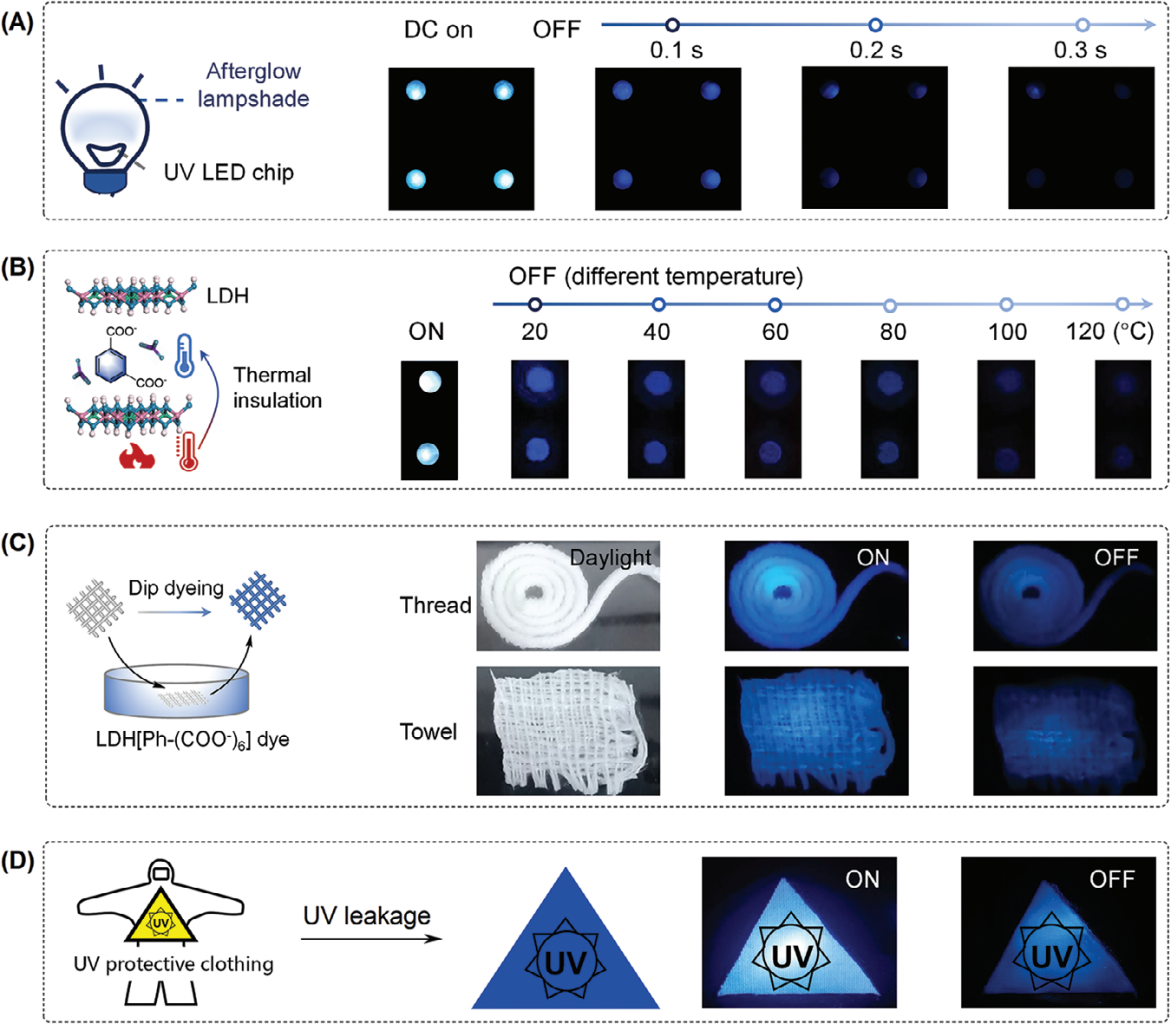
Blue phosphorescence application: A) Schematic structure of photoelectric device and photographs of display taken direct current (DC) on or off for ZnAl‐LDH[Ph‐m(COO^−^)_2_]; B) schematic inllustration for the thermal insulation of LDH and the afterglow images of display with ZnAl‐LDH[Ph‐(COO^−^)_6_] lampshade was heated from 20 to 120 °C by taking DC on or off; C) dip dyeing for cloth by using LDH[Ph‐(COO^−^)_6_] dye; and D) protective clothing for the signing of UV‐leakage.

On the basis of their long lifetime, afterglow materials are appealing for a myriad of applications, such as warning lighting in the dark and near‐zero background bioimaging. Here, applications of ZnAl‐LDH[Ph‐(COO^−^)_n_] were explored based on their unique properties, including deep blue afterglow of the composite, thermal insulation of LDH, UV excitation of resultant afterglow luminescence, and good solution processability. First, deep‐blue lighting is important for full‐color display, the distinguishable deep‐blue afterglow was thus explored for lighting application. For proof‐of‐concept, a photoelectric device was fabricated from a commercial LED chip,^[^ ^15^ ^]^ on which the solution phase LDH[Ph‐m(COO^−^)_2_] was evenly cast to form the lampshade (**Figure** **5**A). In this manner, UV from the LED can be explored as the excitation. Benefiting from the good solution processability of LDH[Ph‐m(COO^−^)_2_], evenly distributed deep‐blue afterglow could be observed after switching off direct current (DC, Figure 5A). Besides, different afterglow digit numbers and paths could be conveniently generated by manipulating the circuitry‐controlled LED array (Figure S25, Supporting Information).

Another intriguing property of LDH is its thermal insulation,^[^ ^16^ ^]^ as evidenced from the by thermo‐gravimetric analysis (Figure S26, Supporting Information, ≈150 °C). The excellent thermal insulation of LDH thus allowed the above fabricated deep‐blue afterglow lighting chip to be emissive at 120 °C (Figure 5B and Figure S27, Supporting Information), indicating that the interlayer of LDH[Ph‐(COO^−^)_n_] exhibited efficient constraints on the molecular motion. Considering that RTP is well‐known for its high temperature‐induced quenching, here the high temperature‐emissive RTP‐based light chip is thus appealing for extreme condition‐based warning lighting.

Last, LDH[Ph‐(COO^−^)_6_] with abundant ‐OH are highly water‐dispersible and can be easily caught by cotton fiber, thus could be explored as luminescent dyes for staining of thread and towel and endow them with blue afterglow emission (Figure 5C). Since the RTP of Ph‐(COO^−^)_6_ could be excited with UV light, the stable staining, and the afterglow emission permitted the design of protective clothing for the signature of UV‐leakage (Figure 5D and Figure S28, Supporting Information).

## 3 Conclusions

In summary, the unique water compatibility and inclusion feature of LDH were explored for harvesting blue phosphorescence from Ph‐(COOH)_n_ in the aqueous phase, resulting in an average lifetime of ZnAl‐LDH[Ph‐(COO^−^)_n_] over 0.1 s. The inclusion of Ph‐(COOH)_n_ into ZnAl‐LDH was confirmed through an anion exchange process, namely NO_3_^−^ out and Ph‐(COO^−^)_n_ in. Through detailed structural analysis, the intermolecular distances of Ph‐(COO^−^)_n_ inside LDH were dependent on the loading amounts. When the loading was lower than 5wt.%, an intermolecular distance longer (>7.0 Å) than the threshold of π–π interaction (3.8 Å) was observed, thus leading to successful isolation of the chromophores and eventually deep blue phosphorescence. Based on the unique properties of LDH, blue phosphoresce materials have great potential to be applied in high‐temperature displays and luminescent dyes. Overall, the good processability of aqueous phase phosphorescence is appealing and may permit a myriad of application, such as full‐color displays and sensors.

## Conflict of Interest

The authors declare no conflict of interest.

## Supporting information



Supporting Information

## Data Availability

The data that support the findings of this study are available from the corresponding author upon reasonable request.
