# Supplementary material for: Enabling Aqueous Phase Long‐lived Deep‐blue Phosphorescence With Layered Double Hydroxide
Source: Adv Sci (Weinh). 2024 Dec 31;12(8):2413896. doi: 10.1002/advs.202413896 (PMC11848578; doi:10.1002/advs.202413896)
Supplement: Supplementary file 1 — Supporting Information [file ADVS-12-2413896-s001.docx]

Supporting Information

**Enabling Aqueous Phase Long-lived Deep-blue Phosphorescence with Layered Double Hydroxide**

*Qian Chen,^a^ Peisheng Cao,^b^ Peng Wu^a, b,^**

^a^Analytical & Testing Center, and ^b^College of Chemistry, Sichuan University, Chengdu, 610064, China

*Corresponding Authors’ E-mail: wupeng@scu.edu.cn

**Table of Contents**

[S1. Research background 3](#_Toc186049801)

[S2. Experimental section 5](#_Toc186049802)

[2.1 Materials 5](#_Toc186049803)

[2.2 Methods 6](#_Toc186049804)

[2.2 Instruments 9](#_Toc186049805)

[S3. Blue RTP of ZnAl-LDH[Ph-(COO^-^)_n_] (n = 1 and 2) in aqueous phase 10](#_Toc186049806)

[S4. Anion exchange 14](#_Toc186049807)

[S5. Mechanism of deep-blue phosphorescence emission 16](#_Toc186049808)

[S6. Universality blue RTP emission of ZnAl-LDH[Ph-(COO^-^)_n_] 18](#_Toc186049809)

[S7. Blue afterglow application 22](#_Toc186049810)

[References 24](#_Toc186049811)

S1. Research background

**Table S1**. Summary of emission wavelength, lifetime and quantum yield of RTP in solution.

| guests | hosts | Emission wavelength (nm) | Lifetime  (ms) | Quantum yield (%) | Ref. |
| --- | --- | --- | --- | --- | --- |
| 1 | Lp | 470 | 632 | 18.04 | ^[1]^ |
| 2 | Lp | 613 | 0.347 | - | ^[2]^ |
| 3 | CD | 480 | 1030 | 3.26 | ^[3]^ |
| 4 | Si NPs | 500 | 2.2 | 22 | ^[4]^ |
| 5 | CB | 500 | 4.33 | 7.58 | ^[5]^ |
| 6 | CB | 527 | 0.53 | - | ^[6]^ |
| 7 | CB | 503 | 0.419 | 2.73 | ^[7]^ |
| 8 | CB | 500 | 0.473 | 5.2 | ^[8]^ |
| 9 | CB | 500 | 4.42 | 1.95 | ^[9]^ |
| 10 | CB | 500 | 0.33 | 7.1 | ^[10]^ |
| 11 | CB | 628 | 0.133 | 12.1 | ^[11]^ |
| 12 | CB | 520 | 2.11 | 25.7 | ^[12]^ |
| 13 | CB | 505 | 1.13 | - | ^[13]^ |
| 14 | CB | 565 | 0.19 | - | ^[14]^ |
| 15 | - | 628 | 0.026 | 6.5 | ^[15]^ |
| 16 | - | 530 | 354 | 1.7 | ^[16]^ |
| 17 | HOF | 527 | 493.1 | 32.09 | ^[17]^ |
| 18 | CD | 500 | 0.96 | 2.8 | ^[18]^ |
| 19 | Lp | 470  560 | 0.37  0.23 | 5.4 | ^[19]^ |
| 20 | CD | 510 | 0.504 | - | ^[20]^ |
| 21 | - | 607 | 3.30 | 3.1 | ^[21]^ |
| Ph-COOH  Ph-m(COOH)_2_  Ph-(COOH)_3_  Ph-(COOH)_4_  Ph-(COOH)_5_  Ph-(COOH)_6_ | LDH | 404  407  424  455  450  443 | 140  100  120  170  120  160 | 2.5  5.88  13.5  37.5  40.0  42.0 | This work |

Abbreviations: Lp, laponite; CB, cucurbiturils; HOF, hydrogen-bonded organic frameworks; CD, Cyclodextrins; LDH, layered double hydroxide.

**Figure S1**. The structure of chromophore molecules (corresponding to Table S1).

S2. Experimental section

2.1 Materials

**Table S2.** The information about the materials used in this work.

| Name | CAS No. | Specification | Supplier |
| --- | --- | --- | --- |
| Zinc nitrate hexahydrate (Zn(NO_3_)_2_·6H_2_O) | 10196-18-6 | AR | Aladdin, Shanghai, China |
| Aluminum nitrate nonahydrate (Al(NO_3_)_3_·9H_2_O) | 7784-27-2 | AR |  |
| Sodium hydroxide (NaOH) | 1310-73-2 | 96% |  |
| Benzoic acid (Ph-COOH) | 68-85-0 | 99.5% |  |
| Isophthalic acid [Ph-m(COOH)_2_] | 121-91-5 | AR |  |
| Trimesic acid [Ph-(COOH)_3_] | 554-95-0 | 98% |  |
| Phthalic acid (o-PA) | 88-99-3 | 99.5% |  |
| p-Phthalic acid (p-PA) | 100-21-0 | 99% |  |
| Pyromellitic acid [Ph-(COOH)_4_] | 89-05-4 | > 98.0% |  |
| Benzenepentacarboxylic acid [Ph-(COOH)_5_] | 1585-40-6 | > 98.0% |  |
| Mellitic acid [Ph-(COOH)_6_] | 517-60-2 | ≥ 98.0% |  |
| Trifluoroacetic acid | 76-05-1 | 0.05% |  |
| Ethyl Alcohol | 64-17-5 | AR | Krohne Chemicals, Chengdu, China |

2.2 Methods

*Synthesis of ZnAl-LDH.* Zn(NO_3_)_2_·6H_2_O (5 mmol) and Al(NO_3_)_3_·9H_2_O (2.5 mmol) were dissolved in 100 mL of water to obtain A solution. NaOH (15 mmol) was dissolved in water solution (100 mL) to form B solution. Afterward, A solution was slowly added into B solution to keep pH about 8.25. The above mixture solution maintained at 60 °C for 24 h in N_2_ atmosphere. Finally, the milky suspension was washed by water and dried at 60 °C overnight to obtain ZnAl-LDH. The mole ration of n(Zn) / n(Al) is 2:1 by inductively coupled plasma mass spectrometry analysis.

*Synthesis of ZnAl-LDH[Ph-(COO^-^)_n_].* First, the as-synthesized ZnAl-LDH (100 mg) was dispersed in 20 mL of ultra-pure water and ethanol solution by ultrasonic. Then, 2 mg of Ph-(COOH)_n_ (n = 1-6) was added above solution and stirred about 12 h at room temperature. Then, ZnAl-LDH[Ph-(COO^-^)_n_] (Loading: 2wt.%) was collected by centrifugation and washed three times. Subsequently, samples were dried by freezing and grinding to form ZnAl-LDH[Ph-(COO^-^)_n_]. The substitution of Ph-m(COOH)_2_ with Phthalic acid and p-Phthalic acid were performed to prepare ZnAl-LDH[Ph-o(COO^-^)_2_] and ZnAl-LDH[Ph-p(COO^-^)_2_], respectively. Besides, the substitution of Ph-m(COOH)_2_ loading with 1wt.%, 3wt.%, 5wt.%, 10wt.%, 15wt.%, 16wt.% and 18wt.% were performed to prepare a series of ZnAl-LDH[Ph-m(COO^-^)_2_]. In this work, the purity of the organic molecules used was characterized with high-performance liquid chromatography (HPLC, acetonitrile-water mixture as the mobile phase, Figure S2). And the corresponding phosphorescence spectra after purification were given (Figure S3).

*Measurement of the concentrations of Ph-m(COO^-^)_2_ and NO_3_^-^ in the interlayer of* *ZnAl-LDH[Ph-m(COO^-^)_2_].* In anion exchange process, the amounts of NO_3_^-^ and Ph-m(COO^-^)_2_ were measured by ion chromatograph and UV-vis absorption spectra, respectively. First, as-obtained ZnAl-LDH[Ph-m(COO^-^)_2_] was dissolved in acid condition (pH < 4, HCl). Then the amounts of Ph-m(COO^-^)_2_ can be detected via UV-vis with characteristic peak at 278 nm (Figure S13). And NO_3_^-^ can be monitored via ion chromatograph.

*Photoluminescence quantum yield.* Absolute quantum yield (*Ф*) of the materials was measured at room temperature using a Fuolog-3 spectrofluorometer equipped with an integrating sphere (IS80, Labsphere).

*Device fabrication.* UV LED chips were purchased from Semiconductor store and acted as excitation sources. ZnAl-LDH[Ph-m(COO^-^)_2_] was well-dispersed in aqueous solution and coated onto the surface of UV LEDs via simple evaporation. Deep-blue afterglow can be observed when LEDs were off. The luminous path is controlled by series parallel.

*Dip dyeing of cloth and Chinese knot model.* LDH[Ph-(COO^-^)_6_] was dispersed in water phase (1 mg/mL). Next, thread and towel were dipped in above solution. After drying, colored cloth was collected. Copper model (good thermal conductivity) with Chinese knot pattern can be acquired from Taobao, and LDH[Ph-(COO^-^)_6_] ink was added into above model.

*Theoretical calculation.* In this work, structure of ZnAl-LDH[Ph-m(COO^-^)_2_] were designed by Material Studio. The geometry relaxation was performed by using Vienna ab-initio software package^[22]^ under Perdew−Burke−Ernzerhof (PBE) functional. The energy cutoff is 400 eV and the *k*-point sets as 1 × 1 × 1. The convergence criterion of atomic power and energy were set to be 0.01 eV/Å and 10^-5^ eV, respectively.

The weak interaction between chromophore molecules were calculated by Gaussian 09^[23]^ via b3lyp with 6-311g (d, p) level in water solvent basic set. The RDG isosurfaces analysis were performed through wave function analysis program Multiwfn^[24]^ and visual molecular dynamics.

**Figure S2**. HPLC chromatographs for Ph-(COOH)_n_ (n = 1-6).

**Figure S3**. Delayed (Δt = 10 ms) emission spectra of pristine and purified Ph-(COOH)_n_ (λ_ex_ = 280 nm).

## 2.2 Instruments

**Table S3**. The instrumental information used in this work.

| Characterization items | Type | Manufacturer |
| --- | --- | --- |
| Phosphorescence spectrum | Fluoromax-4 spectrofluorometer | Horiba Jobin Yvon, USA |
| Photoluminescence spectrum  Phosphorescence lifetime & QY | Fuolog-3 spectrofluorometer | Horiba Jobin Yvon, USA |
| X-ray diffraction | Empyrean | Malvern Panalytical, Netherlands |
| Cs-corrected Transmission electron microscopy | Spectra 300 | Thermo Fisher, USA |
| Inductively coupled plasma mass spectrometry | 7900 | Agilent, USA |
| Element analyzer | Vario EL Cube | Elemental, Germany |
| UV-vis absorption spectra | Lambda-365 | Perkin Elmer, USA |
| Ion chromatograph | CIC-D160 | Sheng Han, China |
| Infrared spectroscopy | Nexus 670 | Thermo Nicolet, USA |
| UV-vis spectrum | UV-3600 | Shimadzu, Japan |

# S3. Blue RTP of ZnAl-LDH[Ph-(COO^-^)_n_] (n = 1 and 2) in aqueous phase


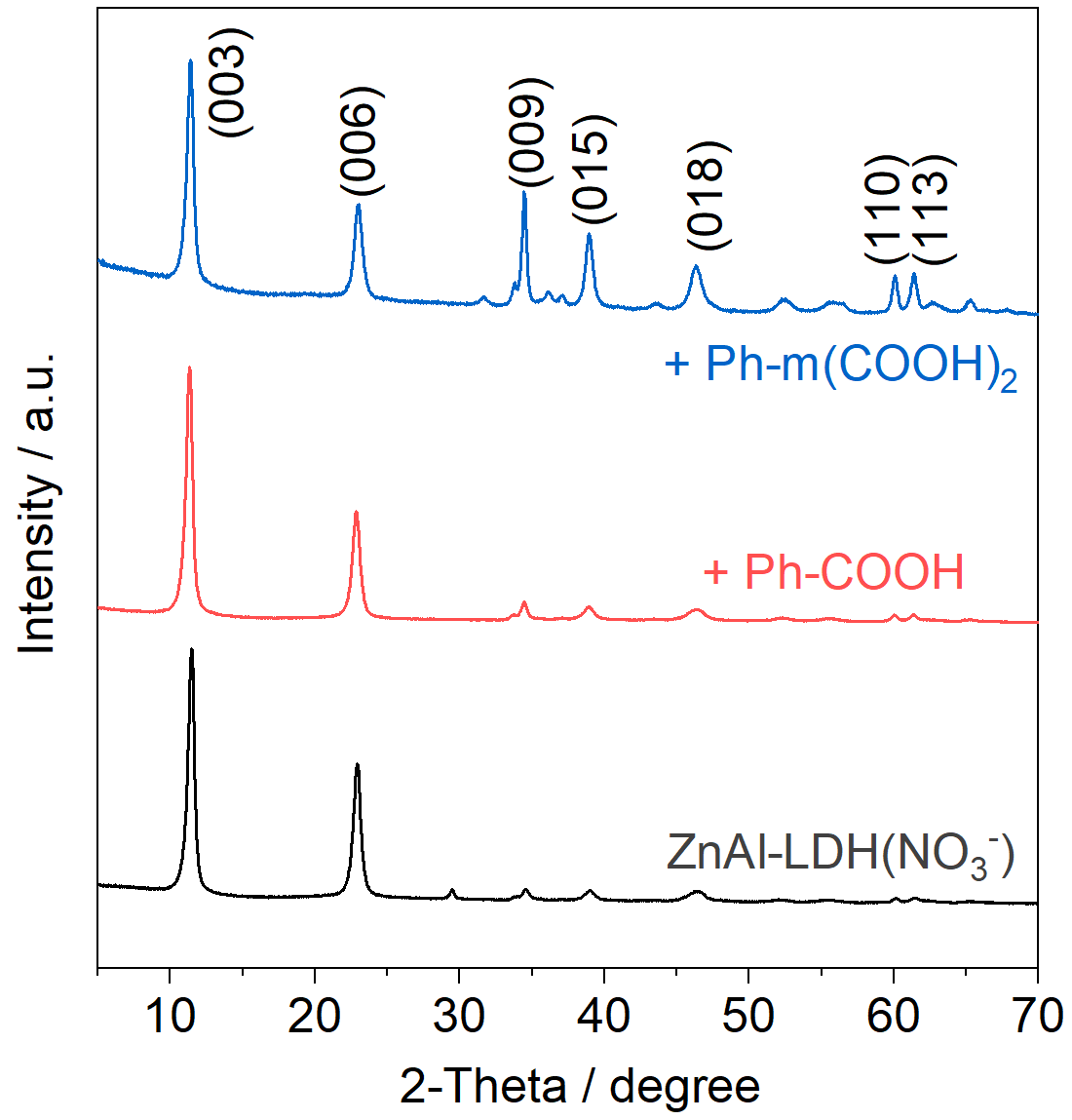


**Figure S4**. X-ray diffraction spectra (XRD) of ZnAl-LDH(NO_3_^-^), ZnAl-LDH(Ph-COO^-^), and ZnAl-LDH[Ph-m(COO^-^)_2_].


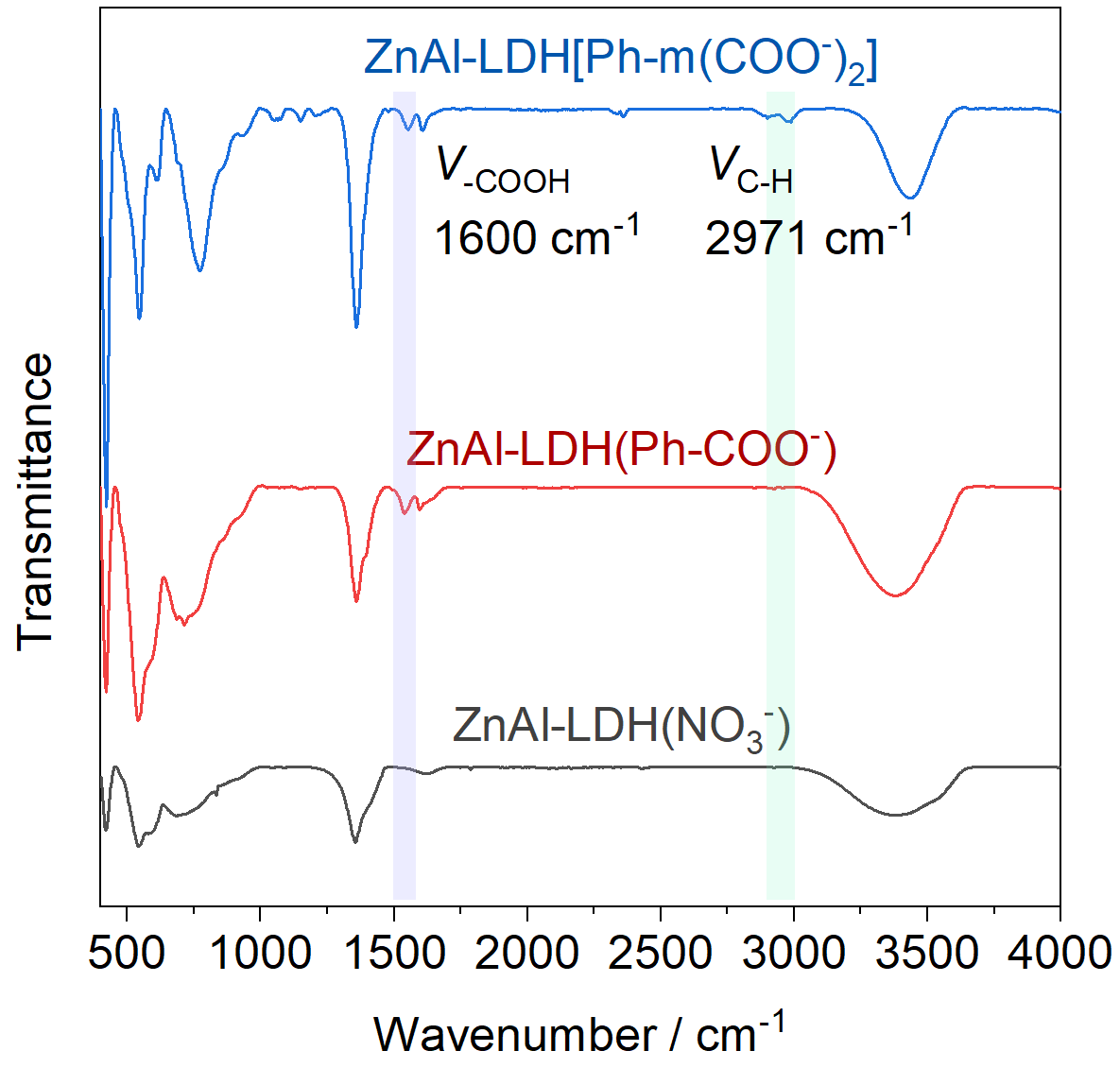


**Figure S5**. Fourier transform infrared (FT-IR) spectra for ZnAl-LDH(NO_3_^-^), ZnAl-LDH(Ph-COO^-^), and ZnAl-LDH[Ph-m(COO^-^)_2_].


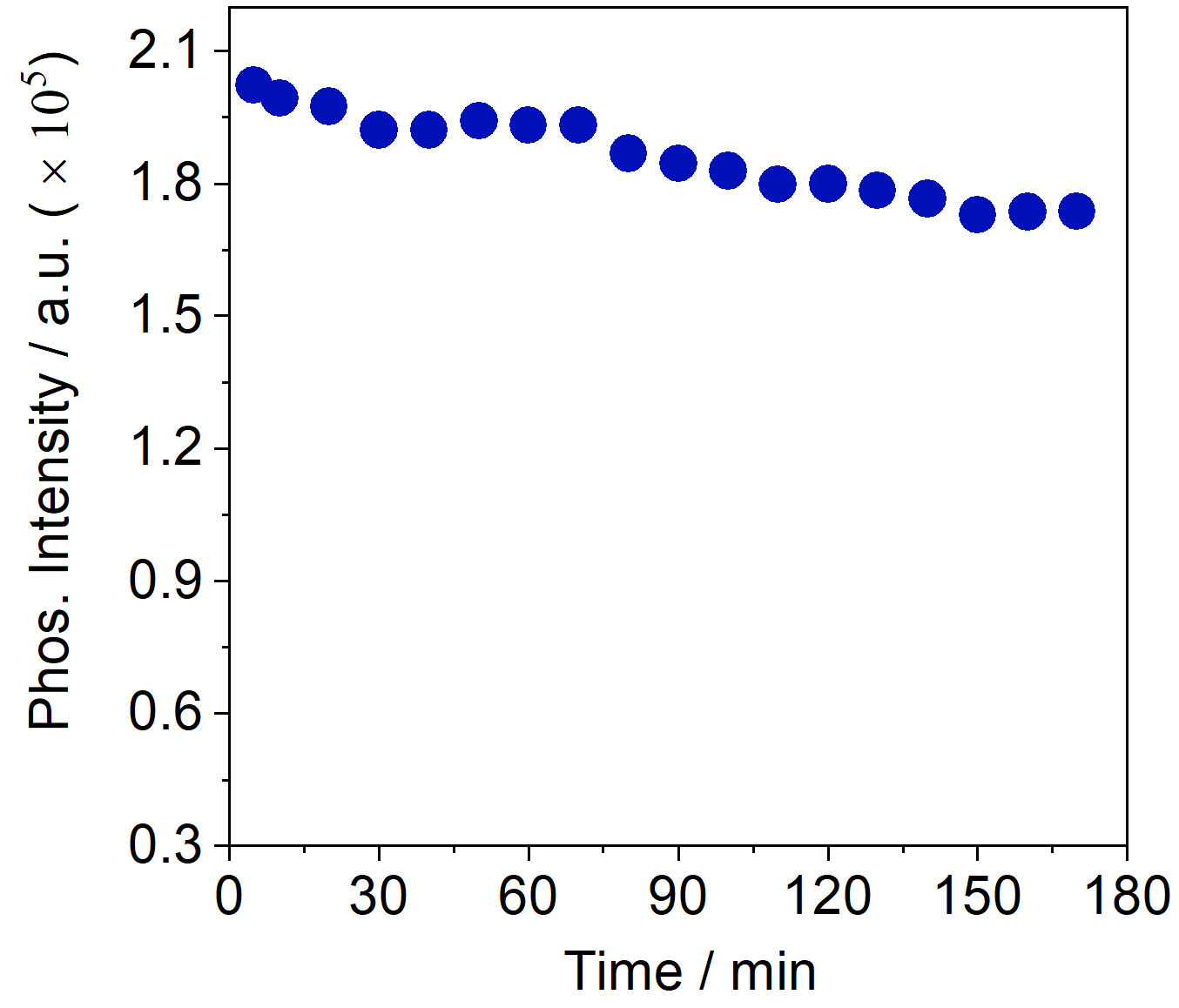


**Figure S6**. Stability of ZnAl-LDH[Ph-m(COO^-^)_2_] in aqueous phase in 180 min (Ex. 280 nm).

**Figure S7.** Steady-state and delayed (Δt = 10 ms) emission spectra of pristine Ph-o(COOH)_2_, LDH[Ph-o(COO^-^)_2_] (A) and pristine Ph-p(COOH)_2_, LDH[Ph-p(COO^-^)_2_] (B) in solid state and water phase (1 mg/mL), respectively, λ_ex_ = 280 nm; (C) CIE 1931 coordinates of phosphorescence emission of LDH[Ph-o(COO^-^)_2_] and LDH[Ph-p(COO^-^)_2_] in water phase under excitation at 280 nm; (D) lifetime of LDH[Ph-o(COO^-^)_2_] (λ_ex_ = 280 nm, λ_em_ = 425 nm) and LDH[Ph-p(COO^-^)_2_] (λ_ex_ = 280 nm, λ_em_ = 424 nm) in water phase (1 mg/mL).


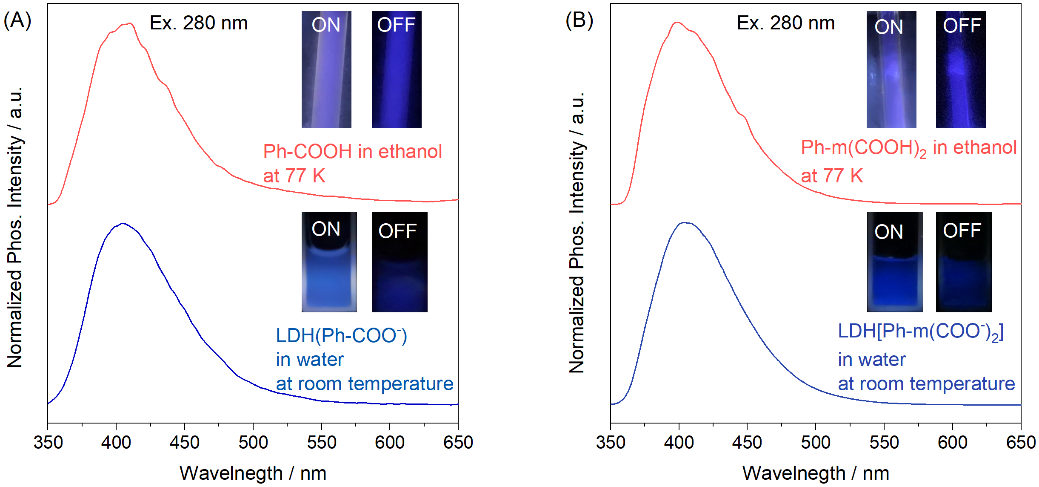


**Figure S8.** Delayed (Δt = 10 ms) emission spectra of (A) pristine Ph-COOH (diluted ethanol solution) at 77 K and LDH(Ph-COO^-^) (aqueous phase, 1 mg/mL) at room temperature, (B) pristine Ph-m(COOH)_2_ (diluted ethanol solution) at 77 K and LDH[Ph-m(COO^-^)_2_] (aqueous phase, 1 mg/mL) at room temperature.

**Figure S9.** Delayed (Δt = 10 ms) emission spectra of (A) LDH(Ph-COO^-^) and (B) LDH[(Ph-COO^-^)_2_] in N_2_ and air (1 mg/mL in water phase).

**Figure S10**. Quantum yields of ZnAl-LDH(Ph-COO^-^) and ZnAl-LDH[Ph-m(COO^-^)_2_] in solid and aqueous phase.

# S4. Anion exchange


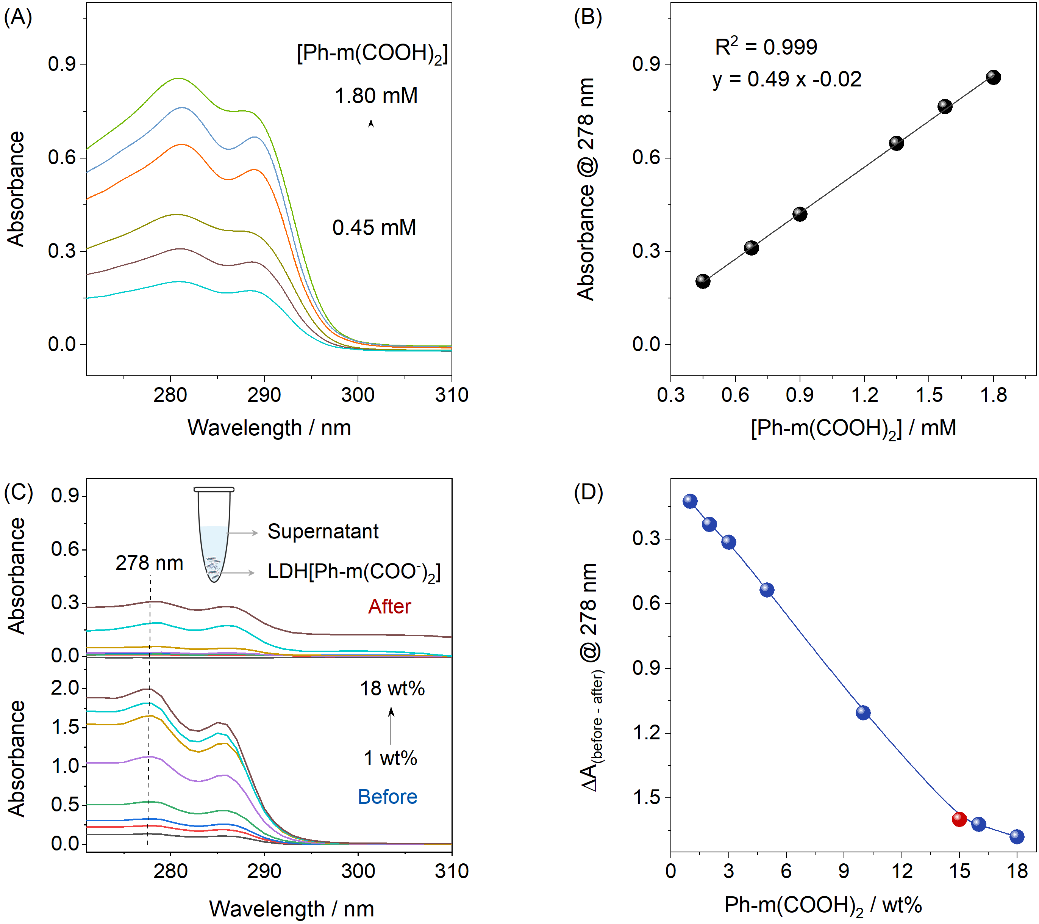


**Figure S11.** Detection of the amounts of Ph-m(COO^-^)_2_ by UV-vis: (A) absorbance spectra of increasing amounts of pristine Ph-m(COO^-^)_2_ solution, and (B) corresponding to linear relationship; (C) absorbance profile in supernatant before and after for anion exchange process; (D) the difference in absorbance (278 nm) before and after anion exchange.


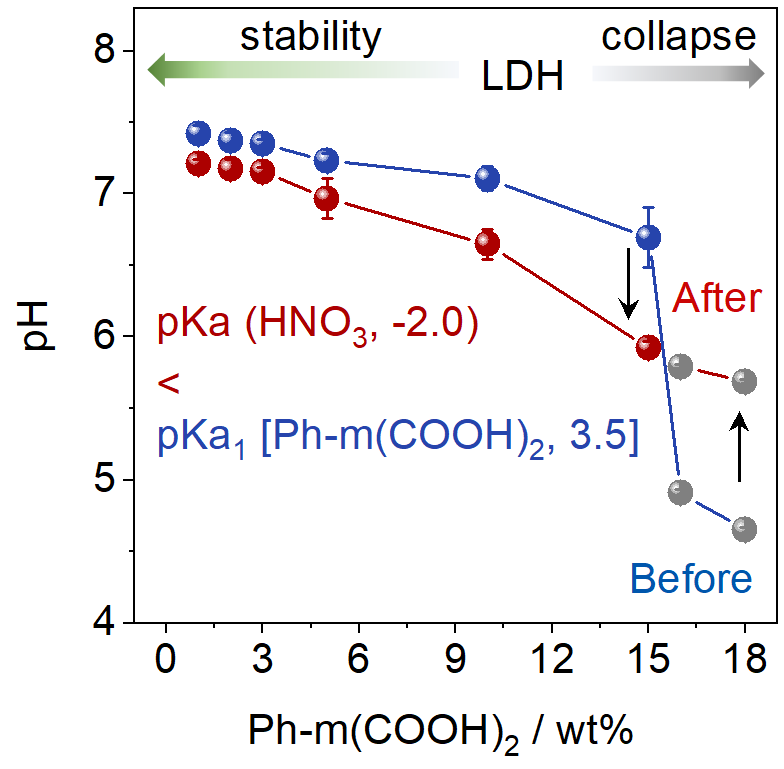


**Figure S12**. pH monitoring in supernatant for anion exchange process with increasing the amounts of Ph-m(COOH)_2_.


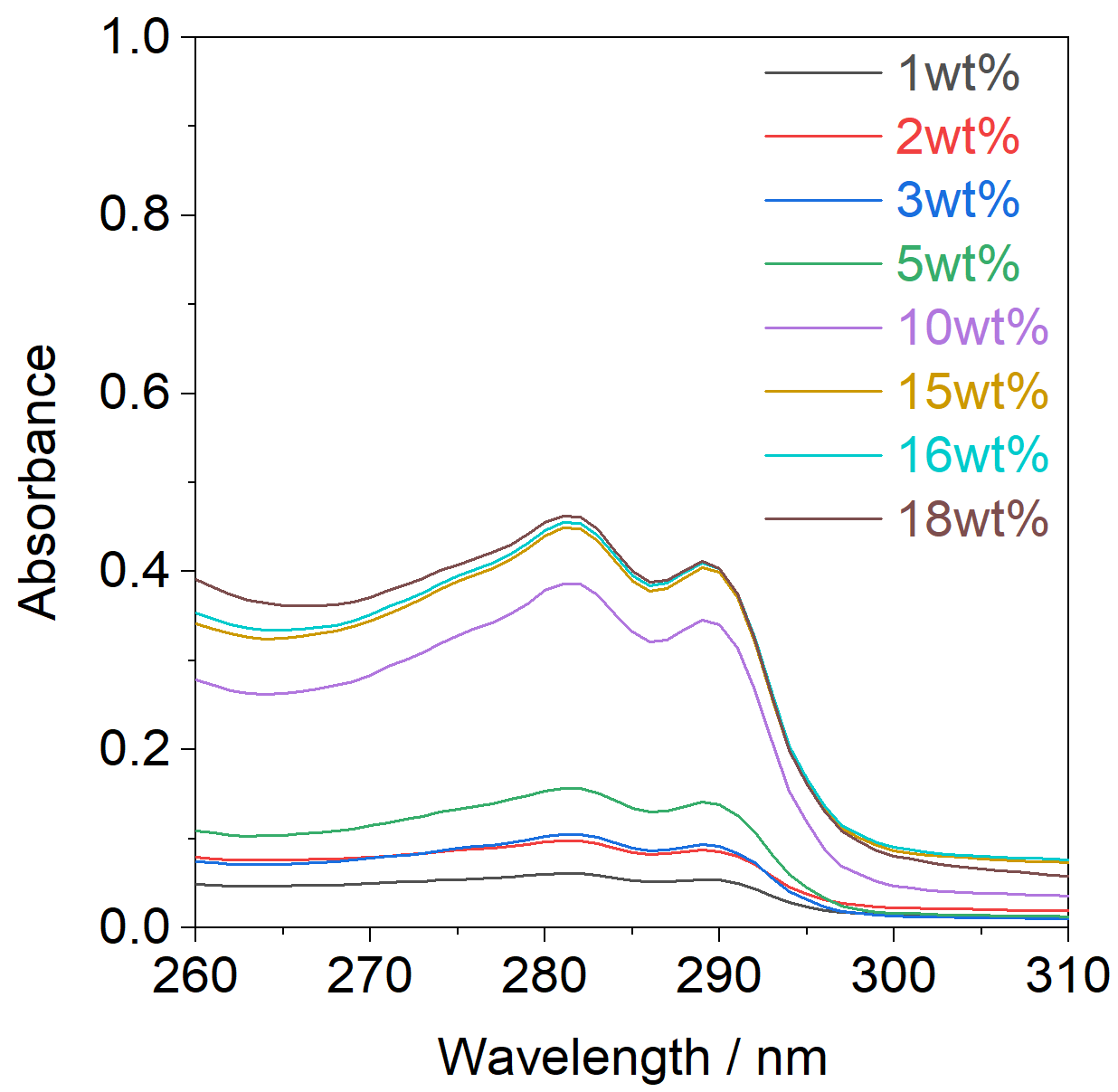


**Figure S13**. The amounts of Ph-m(COO^-^)_2_ in the interlayered of LDH[Ph-m(COO^-^)_2_] with different loading of Ph-m(COOH)_2_.

# S5. Mechanism of deep-blue phosphorescence emission


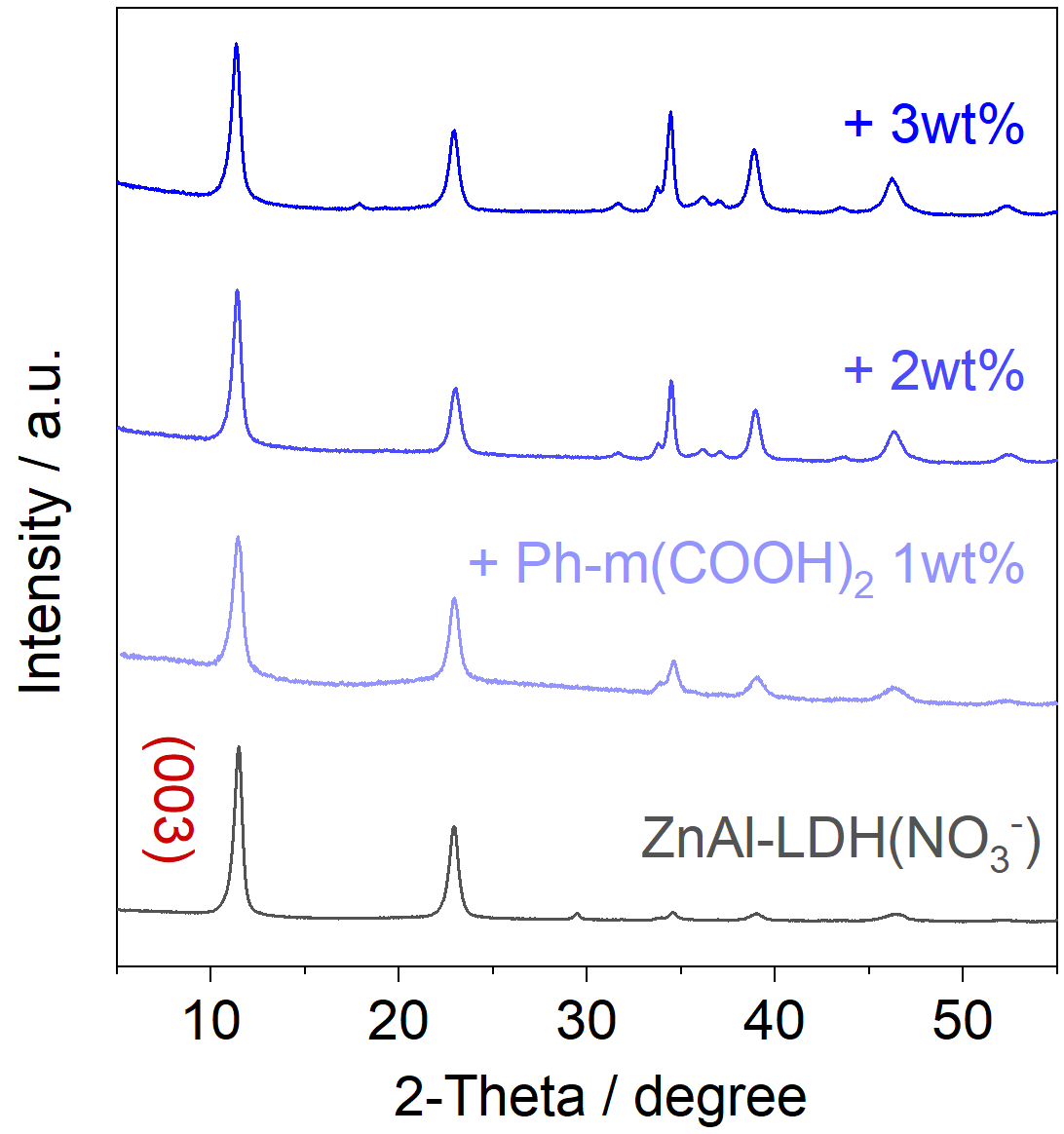


**Figure S14**. XRD of ZnAl-LDH(NO_3_^-^) and LDH[Ph-m(COO^-^)_2_] (loading of Ph-m(COOH)_2_: 1wt%, 2wt% and 3wt%).


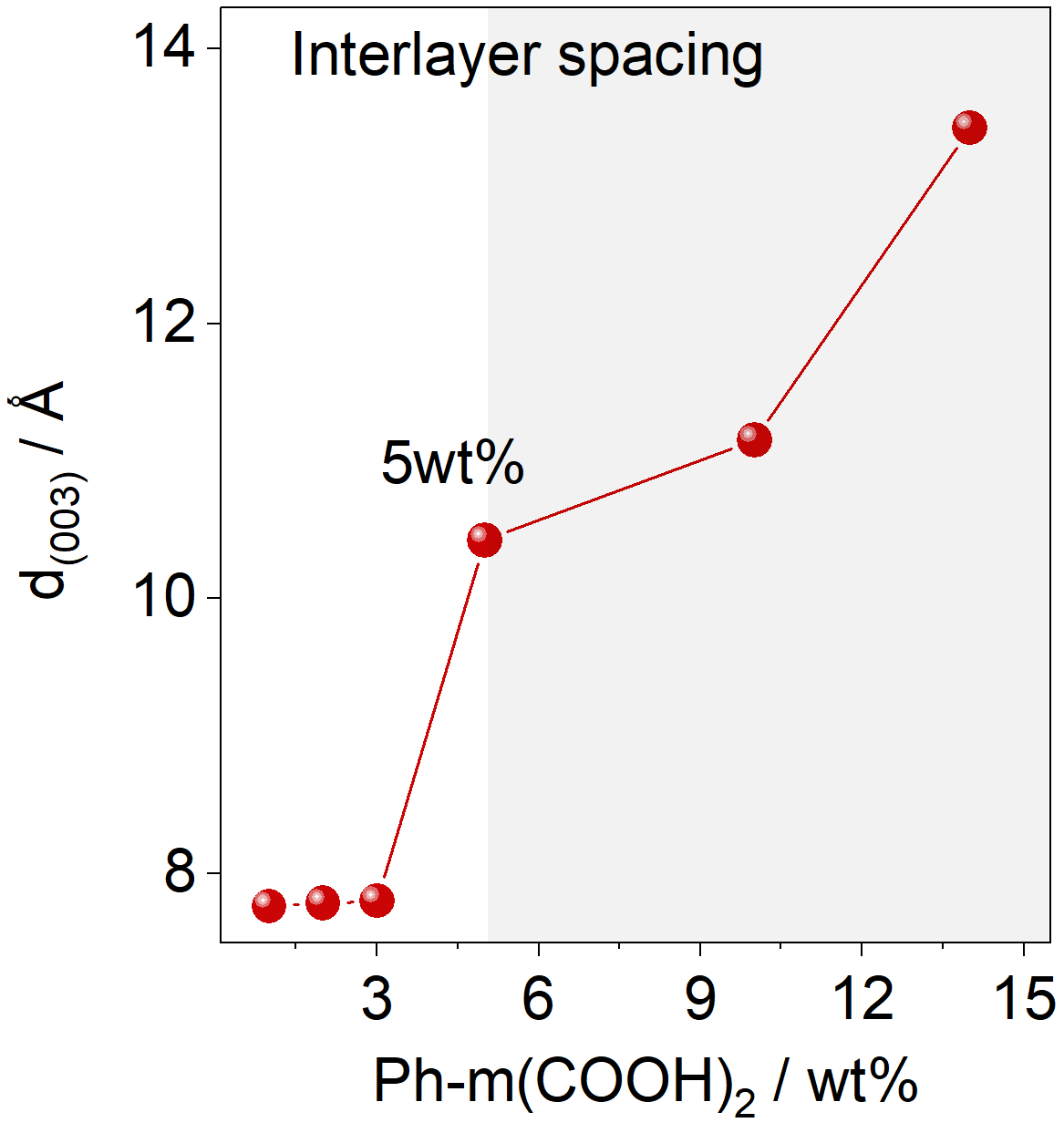


**Figure S15**. The interlayer spacing of LDH[Ph-m(COO^-^)_2_] by Bragg’s law equation calculation (loading of Ph-m(COOH)_2_: 1-15wt%).


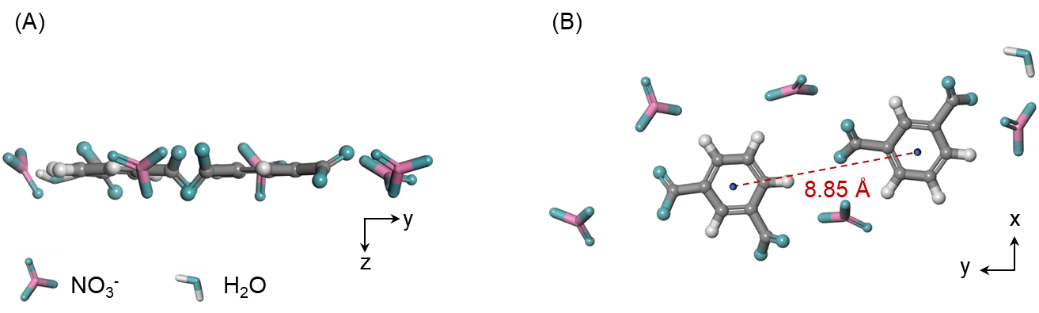


**Figure S16.** The distribution of Ph-m(COO^-^)_2_ and NO_3_^-^ in the interlayered channel of LDH[Ph-m(COO-)_2_] (loading of Ph-m(COOH)_2_: 2wt%): (A) yz planar images; (B) xy planar graph and distance of Ph-m(COO^-^)_2_.


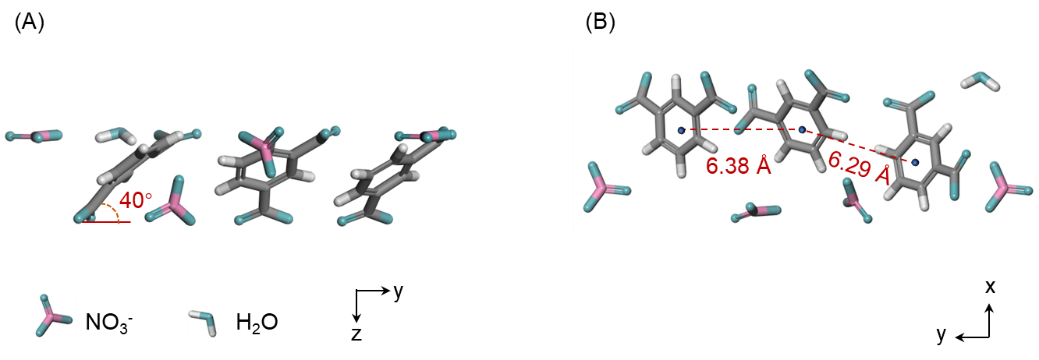


**Figure S17.** The distribution of Ph-m(COO^-^)_2_ and NO_3_^-^ in the interlayered channel of LDH[Ph-m(COO-)_2_] (loading of Ph-m(COOH)_2_: 5wt%): (A) yz planar images; (B) xy planar graph and distance of Ph-m(COO^-^)_2_.


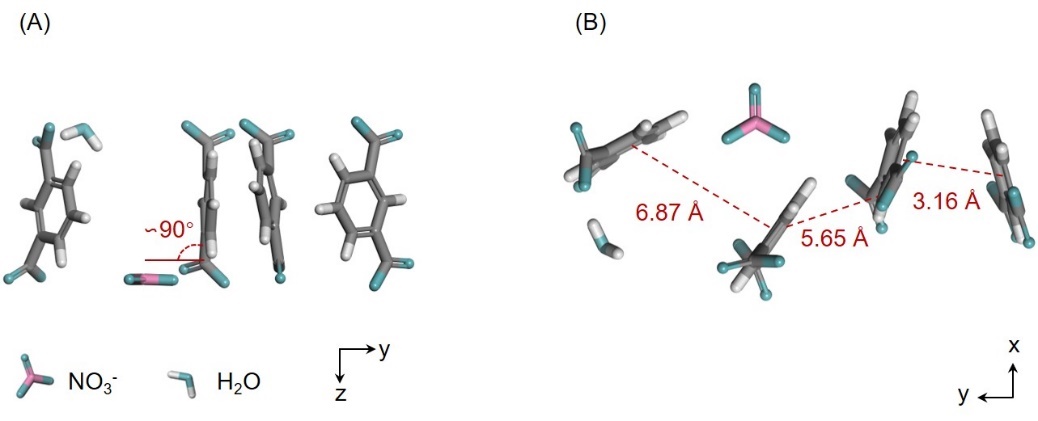


**Figure S18.** The distribution of Ph-m(COO^-^)_2_ and NO_3_^-^ in the interlayered channel of LDH[Ph-m(COO-)_2_] (loading of Ph-m(COOH)_2_: 15wt%): (A) yz planar images; (B) xy planar graph and distance of Ph-m(COO^-^)_2_.

# S6. Universality blue RTP emission of ZnAl-LDH[Ph-(COO^-^)_n_]


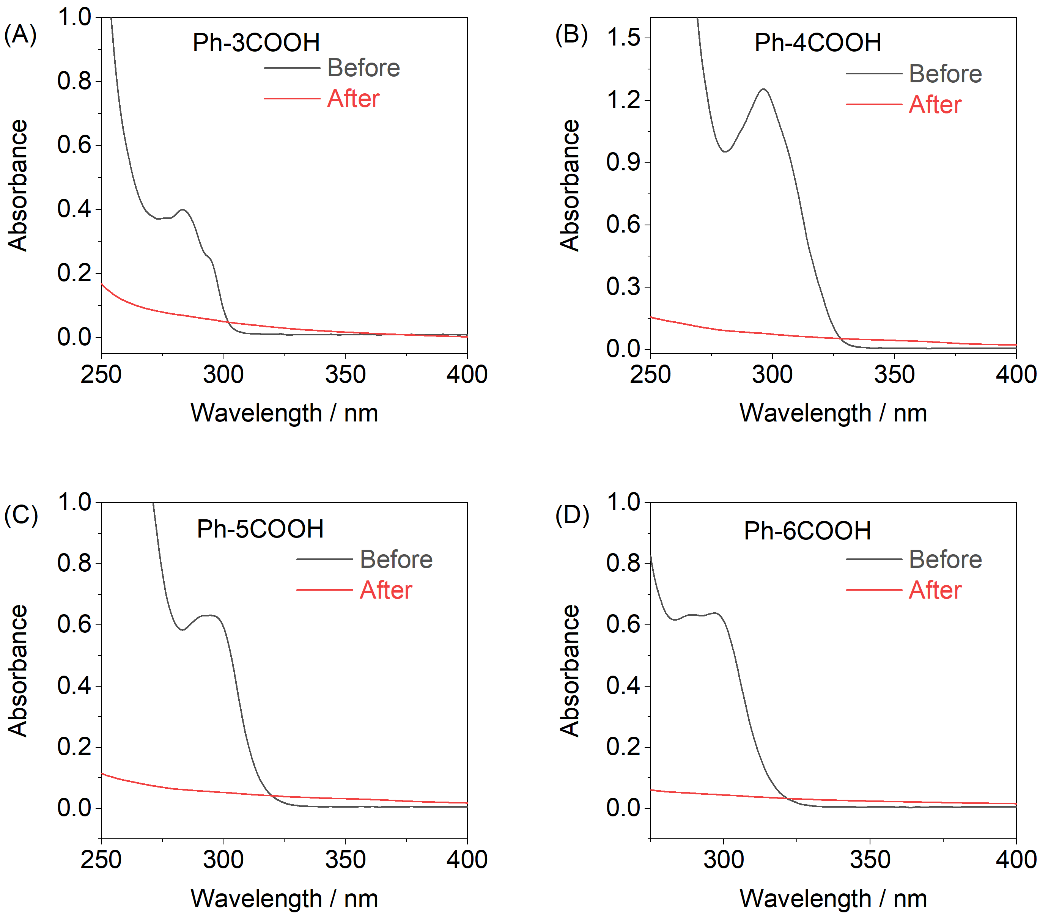


**Figure S19**. Absorption spectra of supernatant before (gray line) and after (red line) reaction for synthesizing ZnAl-LDH[Ph-(COO^-^)_n_] (n = 3-6, loading: 2wt%).


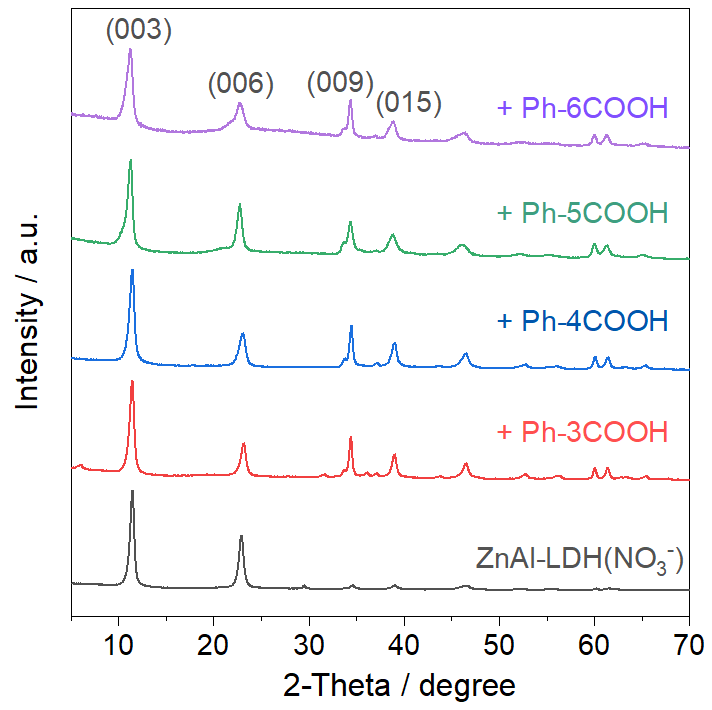


**Figure S20**. XRD spectra of ZnAl-LDH(NO_3_^-^) and ZnAl-LDH[Ph-(COO^-^)_n_] (n = 3-6, loading: 2wt%).


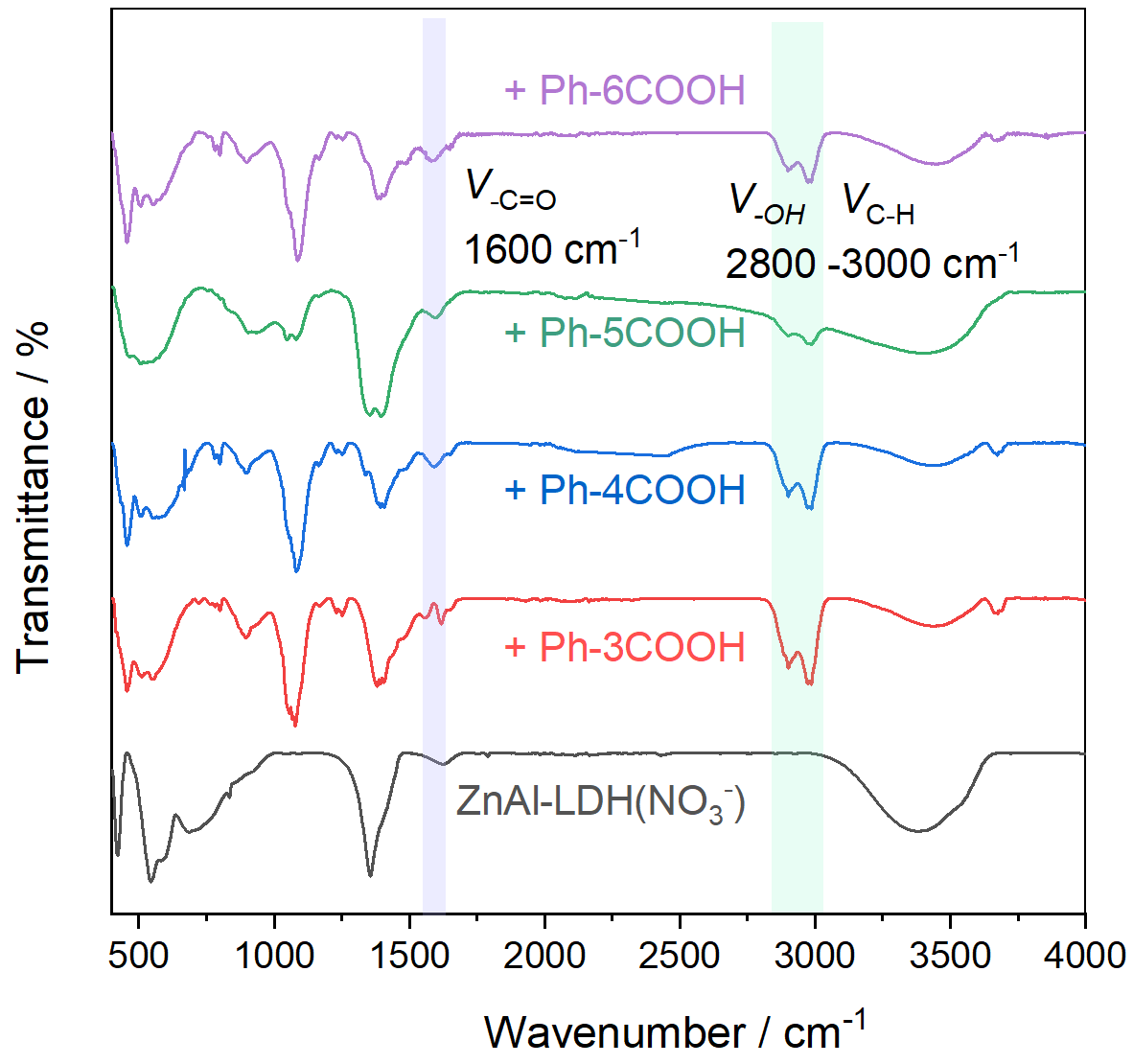


**Figure S21**. FTIR spectra of ZnAl-LDH(NO_3_^-^) and ZnAl-LDH[Ph-(COO^-^)_n_] (n = 3-6, loading: 2wt%).

**Figure S22**. Steady-state and delayed (Δt = 10 ms) emission spectra of pristine Ph-(COOH)_n_ and ZnAl-LDH[Ph-(COO^-^)_n_] in aqueous phase (1mg/mL, λ_ex_ = 280 nm).


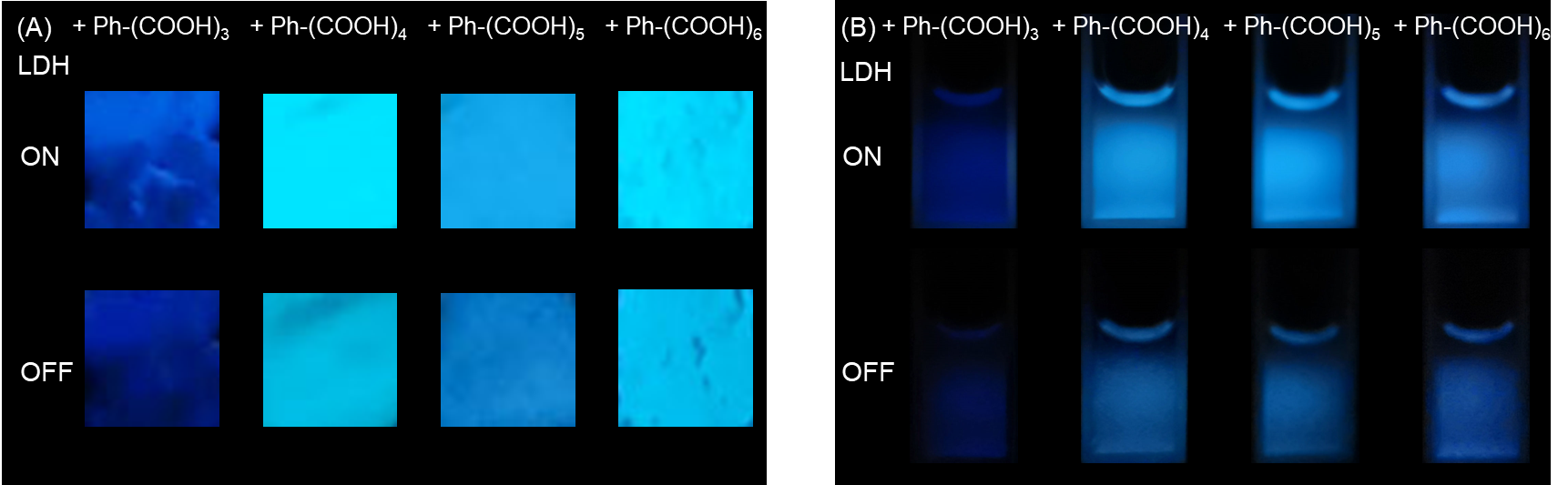


**Figure S23**. Afterglow photos of ZnAl-LDH[Ph-(COO^-^)_n_] in solid and aqueous phase (1mg/mL, λ_ex_ = 280 nm).


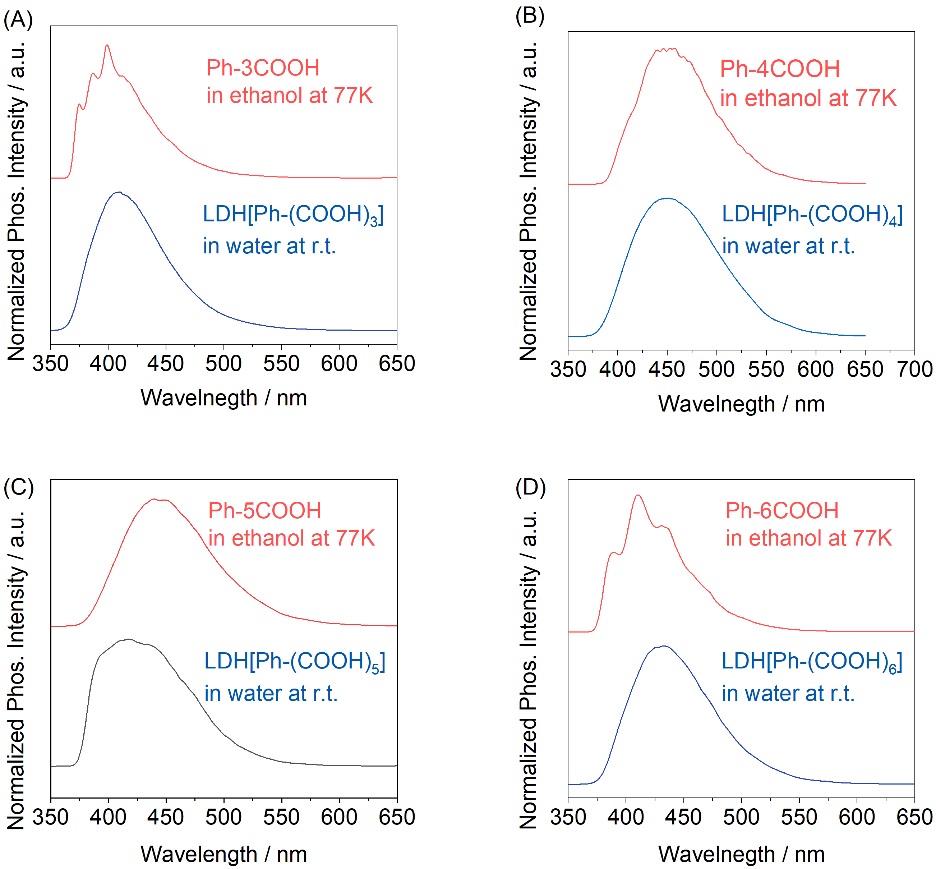


**Figure S24.** Delayed (Δt = 10 ms) emission spectra of Ph-n(COOH) in ethanol solution at 77 K and LDH[Ph-(COO^-^)_n_] (n = 3-6) in water phase(1mg/mL) at room temperature: (A) Ph-3COOH, (B) Ph-4COOH, (C) Ph-5COOH and (D) Ph-6COOH.

**Table S4**. The photophysical data of all materials in this work.

|  | Pristine | | LDH[Ph-(COO^-^)_n_] | | |
| --- | --- | --- | --- | --- | --- |
|  | λ_Phos_ / nm | τ_Phos_ / s | Condition | λ_Phos_ / nm | τ_Phos_ / s |
| Ph-COOH | 506 | 0.25 | Solid | 410 | 0.14 |
|  |  |  | Water | 404 | 0.13 |
| Ph-o(COOH)_2_ | 533 | 0.092 | Solid | 431 | 0.22 |
|  |  |  | Water | 425 | 0.20 |
| Ph-m(COOH)_2_ | 524 | 0.61 | Solid | 416 | 0.10 |
|  |  |  | Water | 407 | 0.11 |
| Ph-p(COOH)_2_ | 518 | 0.25 | Solid | 437 | 0.12 |
|  |  |  | Water | 424 | 0.16 |
| Ph-(COOH)_3_ | 533 | 0.20 | Solid | 425 | 0.12 |
|  |  |  | Water | 424 | 0.12 |
| Ph-(COOH)_4_ | 542 | 0.57 | Solid | 458 | 0.17 |
|  |  |  | Water | 455 | 0.17 |
| Ph-(COOH)_5_ | 518 | 0.080 | Solid | 443 | 0.11 |
|  |  |  | Water | 450 | 0.12 |
| Ph-(COOH)_6_ | 509 | 0.065 | Solid | 464 | 0.17 |
|  |  |  | Water | 443 | 0.16 |

# S7. Blue afterglow application


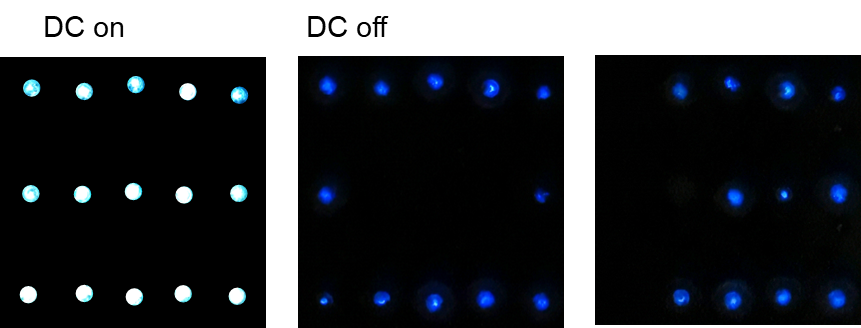


**Figure S25.** Afterglow display of different paths by manipulating series parallel.

**Figure S26.** Thermo-gravimetric analysis for ZnAl-LDH.


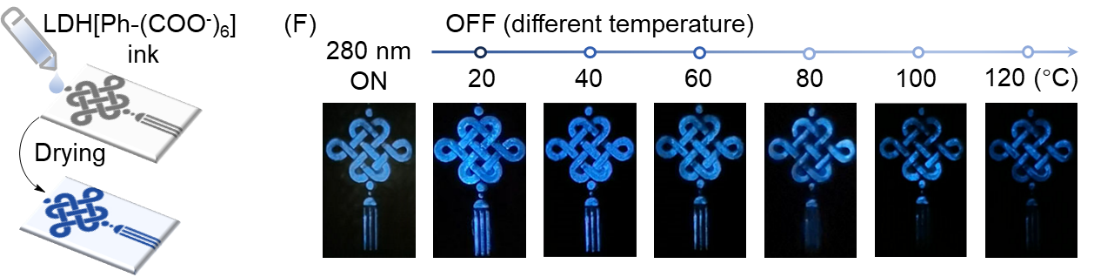


**Figure S27.** Copper matrix with ZnAl-LDH[Ph-(COO^-^)_6_] was heated from 20 to 120 °C and afterglow images under 280 nm UV.


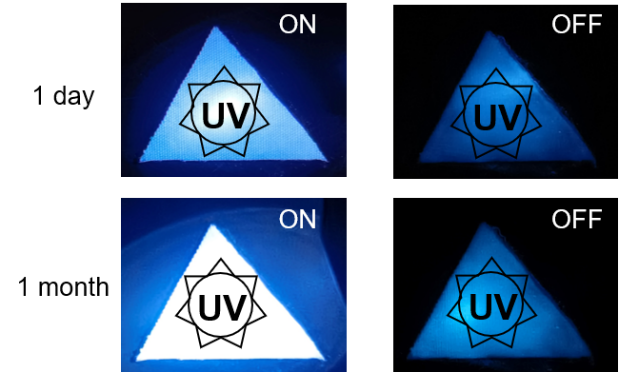


**Figure S28.** Dip dyeing for cloth about 1day and one month.

References

[1] Y. Deng, P. Li, J. Li, D. Sun, H. Li, *ACS Appl. Mater. Interfaces* **2021**, 13, 14407.

[2] S. Kuila, K. V. Rao, S. Garain, P. K. Samanta, S. Das, S. K. Pati, M. Eswaramoorthy, S. J. George, *Angew. Chem. Int. Ed.* **2018**, 57, 17115.

[3] D. Li, Z. Liu, M. Fang, J. Yang, B. Z. Tang, Z. Li, *ACS Nano* **2023**, 17, 12895.

[4] T. He, W.-J. Guo, Y.-Z. Chen, X.-F. Yang, C.-H. Tung, L.-Z. Wu, *Aggregate* **2023**, 4, e250.

[5] W.-L. Zhou, Y. Chen, Q. Yu, H. Zhang, Z.-X. Liu, X.-Y. Dai, J.-J. Li, Y. Liu, *Nat. Commun.* **2020**, 11, 4655.

[6] X. Ma, J. Cao, Q. Wang, H. Tian, *Chem. Commun.* **2011**, 47, 3559.

[7] X.-K. Ma, Y.-M. Zhang, Q. Yu, H. Zhang, Z. Zhang, Y. Liu, *Chem. Commun.* **2021**, 57, 1214.

[8] C. Xu, X. Lin, W. Wu, X. Ma, *Chem. Commun.* **2021**, 57, 10178.

[9] W.-L. Zhou, W. Lin, Y. Chen, X.-Y. Dai, Z. Liu, Y. Liu, *Chemical Science* **2022**, 13, 573.

[10] S. K. Bhaumik, S. K. Panda, S. Banerjee, *Chem. Commun.* **2023**, 59, 10396.

[11] X.-K. Ma, W. Zhang, Z. Liu, H. Zhang, B. Zhang, Y. Liu, *Adv. Mater.* **2021**, 33, 2007476.

[12] W.-W. Xing, H.-J. Wang, Z. Liu, Z.-H. Yu, H.-Y. Zhang, Y. Liu, *Adv. Opt. Mater.* **2023**, 11, 2202588.

[13] M. Huo, X.-Y. Dai, Y. Liu, *Angew. Chem. Int. Ed.* **2021**, 60, 27171.

[14] J. Wang, Z. Huang, X. Ma, H. Tian, *Angew. Chem. Int. Ed.* **2020**, 59, 9928.

[15] X.-F. Wang, H. Xiao, P.-Z. Chen, Q.-Z. Yang, B. Chen, C.-H. Tung, Y.-Z. Chen, L.-Z. Wu, *J. Am. Chem. Soc.* **2019**, 141, 5045.

[16] X. Zhen, Y. Tao, Z. An, P. Chen, C. Xu, R. Chen, W. Huang, K. Pu, *Adv. Mater.* **2017**, 29, 1606665.

[17] W. Luo, J. Zhou, Y. Nie, F. Li, S. Cai, G. Yin, T. Chen, Z. Cai, *Adv. Funct. Mater.* , n/a, 2401728.

[18] D. Li, F. Lu, J. Wang, W. Hu, X.-M. Cao, X. Ma, H. Tian, *J. Am. Chem. Soc.* **2018**, 140, 1916.

[19] S. Garain, B. C. Garain, M. Eswaramoorthy, S. K. Pati, S. J. George, *Angew. Chem. Int. Ed.* **2021**, 60, 19720.

[20] F.-F. Shen, Y. Chen, X. Dai, H.-Y. Zhang, B. Zhang, Y. Liu, Y. Liu, *Chemical Science* **2021**, 12, 1851.

[21] X.-F. Wang, W.-J. Guo, H. Xiao, Q.-Z. Yang, B. Chen, Y.-Z. Chen, C.-H. Tung, L.-Z. Wu, *Adv. Funct. Mater.* **2020**, 30, 1907282.

[22] G. Kresse, J. Furthmüller, Comput. Mater. Sci. 1996, 6, 15.

[23] M. J. Frisch, G. W. Trucks, H. B. Schlegel, G. E. Scuseria, M. A. Robb, J. R. Cheeseman, G. Scalmani, V. Barone, G. A. Petersson, H. Nakatsuji, M. Caricato, X. Li, H. P. Hratchian, A. F. Izmaylov, J. Bloino, G. Zheng, J. L. Sonnenberg, M. Hada, M. Ehara, K. Toyota, R. Fukuda, J. Hasegawa, M. Ishida, T. Nakajima, Y. Honda, O. Kitao, H. Nakai, T. Vreven, J. A. Montgomery Jr., J. E. Peralta, F. Ogliaro, M. J. Bearpark, J. J. Heyd, E. N. Brothers, K. N. Kudin, V. N. Staroverov, R. Kobayashi, J. Normand, K. Raghavachari, A. P. Rendell, J. C. Burant, S. S. Iyengar, J. Tomasi, M. Cossi, N. Rega, J. M. Millam, M. Klene, J. E. Knox, J. B. Cross, V. Bakken, C. Adamo, J. Jaramillo, R. Gomperts, R. E. Stratmann, O. Yazyev, A. J. Austin, R. Cammi, C. Pomelli, J. W. Ochterski, R. L. Martin, K. Morokuma, V. G. Zakrzewski, P. Salvador, J. J. Dannenberg, S. Dapprich, A. D. Daniels, O. Farkas, J. B. Foresman, D. J. Fox, Wallingford, CT 2009.

[24] T. Lu, F. Chen, J. Comput. Chem. 2012, 33, 580.
